# Genome-Wide Identification and Characterization of the PP2C Gene Family in *Gossypium barbadense* Reveals Potential Candidates for Breeding Improved Stress Resistance, Fiber Character, and Early Maturing Cotton Varieties

**DOI:** 10.3390/cimb47120977

**Published:** 2025-11-24

**Authors:** Nan Zhao, Weiran Wang, Zixin Zhou, Meng Wang, Caixia Li, Lingfang Ran, Yaohua Li, Jianping Li, Jiahui Zhu, Zhiqing Liu, Yifan Wang, Yahui Deng, Jing Yang, Alifu Aierxi, Jie Kong

**Affiliations:** Cotton Research Institute, Xinjiang Uyghur Autonomous Region Academy of Agricultural Sciences (National Cotton Engineering Technology Research Center), Urumqi 830091, China; nan_zhao@cau.edu.cn (N.Z.);

**Keywords:** PP2C gene family, *Gossypium barbadense*, stress resistance, fiber character, early maturity

## Abstract

The protein phosphatase 2C (PP2C) gene family plays vital roles in plant growth and stress responses, yet remains inadequately characterized in cotton, particularly in *Gossypium barbadense* renowned for its superior fiber quality. Here, we identified 152 GbPP2Cs in *Gossypium barbadense* through genome-wide analysis and comparative genomics with three related cotton species (*G. arboreum*, *G. raimondii*, and *G. hirsutum*), identifying 105, 105, and 204 GbPP2Cs, respectively. GbPP2Cs show uneven chromosomal distribution with notable clustering on A05, evolutionary conservation in gene structure and motif composition, and predominant nuclear/chloroplast localization. Phylogenetic analysis classified them into 15 subfamilies showing conserved evolution. Protein enrichment revealed 15 GbPP2Cs involved in mitogen-activated protein kinase (MAPK) and hormone signaling pathways. Expression profiling revealed distinct members responsive to biotic/abiotic stresses, fiber development stages, and maturity. Notably, we discovered potential pleiotropic regulators including two genes (*Gbar_D13G012000* and *Gbar_A13G012360*) co-regulating lint percentage and disease resistance, *GbAIP1* coordinating fiber length-strength trade-off, and *GbPP2C59* as a maturity negative candidate. These findings provide valuable genetic resources for cotton improvement.

## 1. Introduction

Sea Island cotton (*Gossypium barbadense* L.) is renowned for producing the finest quality natural fiber, prized in the textile industry for its exceptional length, strength, and fineness [1]. In China, its cultivation is predominantly confined to Xinjiang, where production is severely threatened by a combination of abiotic stresses, including salinity and drought [2], and biotic challenges such as *Fusarium wilt* [3]. Enhancing the resilience of *G. barbadense* without compromising its superior fiber quality and yield is therefore a critical breeding objective. Unraveling the genetic basis of these complex traits is essential for designing strategic crop improvement programs.

The type 2C protein phosphatase (PP2C) gene family, which is evolutionarily conserved and ubiquitous in virtually all organisms except viruses, is the most abundant among dephosphorylating enzymes in plants [4]. In *Arabidopsis thaliana*, the PP2C family is expanded to 76 putative members [5,6,7]. Most PP2C genes in *A. thaliana* show close relationships with those from other plant species, clustering together into distinct clades [5]. In plants, most PP2C phosphatases possess a conserved catalytic domain at the C-terminus and a variable extension region at the N-terminus where they contain sequences associated with intracellular signaling, such as transmembrane domains and kinase interaction motifs, that confer diverse biological functions to PP2Cs [6]. For instance, in *Arabidopsis*, the abscisic acid insensitive (ABI)-type PP2C proteins contain an EF-hand motif extending from the N-terminus to the catalytic region, which binds Ca^2+^ and regulates the enzymatic activity controlled by the C-terminal catalytic domain [8].

The PP2C gene family plays versatile roles in plant stress adaptation and developmental regulation [9]. In stress responses, PP2Cs often modulate key signaling pathways. *Arabidopsis* HAI phosphatases (HAI1, HAI2, and HAI3) interact with mitogen-activated protein kinase 3/6 (MPK3/6) to mediate abscisic acid (ABA)-dependent immune suppression [10]. *G. hirsutum* GhDRP1s function as a negative regulator of drought tolerance through abscisic acid (ABA) and flavonoid pathway [11]. *G. barbadense* GbPP2C80s negatively modulate the resistance to wilt diseases through MPK3 and reactive oxygen species (ROS) pathways [3]. Such regulatory roles extend to other species, including *Apocynum* and sugarcane, where specific PP2Cs respond to salt or cold stress [12,13]. PP2Cs also participate in developmental processes: *SlPP2C* silencing delays senescence in tomato [14], and BjuPP2C52 interacts with transcription factors to control flowering time in *Brassica juncea* [15], collectively highlighting the functional diversity and conservation of this gene family.

Given their potential to influence stress adaptation and developmental processes, we hypothesized that the PP2C gene family represents a valuable genetic resource for improving both resistance, fiber, and maturity in *G. barbadense*. Although the PP2C gene family has been systematically characterized in *G. hirsutum* [16], a comprehensive analysis in its allotetraploid relative, *G. barbadense*, renowned for superior fiber quality but characterized by a distinct genetic background, has been lacking. This study provides a comparative evolutionary analysis of the PP2C family across four *Gossypium* species and, importantly, integrates multi-omics data to identify members associated with key agronomic traits, including stress resilience, elite fiber properties, and early maturity, thereby offering novel insights and genetic resources for *G. barbadense* improvement.

## 2. Materials and Methods

### 2.1. Identification of the PP2C Gene Family in Four Cotton Genomes

The whole-genome sequences of four cotton species, *G. barbadense* (HAU), and *G. hirsutum* (HAU), *G. arboreum* (CRI), and *G. raimondii* (JGI), were downloaded from the CottonGen database (https://www.cottongen.org/ (accessed on 1 September 2025)) [17]. A total of 76 known *Arabidopsis* PP2C protein sequences were retrieved from the TAIR website (http://www.Arabidopsis.org/ (accessed on 2 September 2025)) and used as queries to perform local BLASTP and BLASTN searches against the genomic databases of the four cotton species. An E-value cutoff of 1 × 10^−5^ was applied to identify initial candidate PP2C genes. To further verify the presence of the conserved PP2C domain, all candidate protein sequences were subjected to a second screening using the Hidden Markov Model (HMM) profiles of the PP2Cc domain (PF00481 and PF07228), which were obtained from the Pfam database (http://pfam.xfam.org/ (accessed on 4 September 2025)) [18]. This search was performed locally using the HMMER 3.4 with default E-value parameters.

### 2.2. Chromosomal Distribution, Gene Structure, Conserved Motifs of PP2C Genes

The chromosomal locations of all identified PP2C genes were mapped onto the four cotton genomes using TBtools v2.056 [19]. Conserved motifs were identified using the Multiple Expectation Maximization for Motif Elicitation (MEME) version 5.5.8 website (https://meme-suite.org/meme/tools/meme (accessed on 6 September 2025)), with the maximum number of motifs set to 10. Conserved protein domains were annotated using NCBI conserved domain database (CDD) website (https://www.ncbi.nlm.nih.gov/Structure/cdd (accessed on 8 September 2025)). All resulting gene structures, motifs, and domains were visualized using TBtools v2.056 [19].

### 2.3. Physicochemical Properties and Subcellular Localization of PP2C Family Members

Physicochemical properties, including the molecular weight (MW) and theoretical isoelectric point (pI), of the identified PP2C proteins were predicted using the Compute pI/Mw tool on the ExPASy online platform (http://web.expasy.org/compute_pi/ (accessed on 10 September 2025)) [20]. Subcellular localization predictions were performed using the WoLF PSORT tool (https://www.genscript.com/wolf-psort.html (accessed on 14 September 2025)) and TargetP-2.0 software (https://services.healthtech.dtu.dk/services/TargetP-2.0/ (accessed on 16 September 2025)), which analyzes amino acid sequences for the presence of targeting signals indicative of specific cellular compartments.

### 2.4. Phylogenetic and Functional Protein Network Analyses of PP2C Family Members

The phylogenetic tree was inferred using Maximum Likelihood (ML) method in MEGA 11, following multiple sequence alignment of the PP2C proteins with ClustalW [21]. The Poisson correction model was selected, and the robustness of the tree topology was assessed with 1000 bootstrap replicates. The final phylogenetic tree was visualized and annotated using the online tool Interactive Tree of Life (iTOL) v6.7.6 (https://itol.embl.de/ (accessed on 15 October 2025)). Protein–protein interactive network prediction, Gene Ontology (GO) and Kyoto Encyclopedia of Genes and Genomes (KEGG) enrichment analyses were performed using STRING version 12.0 (https://cn.string-db.org/ (accessed on 13 October 2025)).

### 2.5. Expression Analysis of PP2C Family Members

For biotic-stress-related genes, fold-change values of differential expression genes from hypocotyls of *Fusarium oxysporum* f. sp. *vasinfectum* (*Fov*)-infected *Fov7*_knockout (KO)#5 versus J668 at 5 and 10 days post-inoculation (dpi) were retrieved [22]. For abiotic-stress-related genes, transcript per lilobase per million mapped reads (TPM) values of PP2C genes from *G. barbadense* cultivar H7124 treated by cold, heat, drought, and salt for 0, 1, 3, 6, 12, 24 h were also retrieved from the CottonMD database (https://yanglab.hzau.edu.cn/CottonMD (accessed on 17 September 2025)) [23]. For fiber-quality-related genes, fragments per kilobase of exon per million reads mapped (FPKM) values of PP2C genes from different fiber developmental stages of *G. barbadense* cultivar XH37 (high fiber strength), XH58 (long fiber), XH33 (low lint percentage), LuoSaiNa (low fiber strength) and AShi (short fiber and high lint percentage) were retrieved [1]. For early-maturation-related genes, FPKM values of PP2C genes from shoot apical meristem (SAM) and flower mixture of early- and late-maturation *G. barbadense* cultivars were retrieved (unpublished). Differentially expressed genes were identified using adjusted *p*-value (FDR) < 0.05 and |log_2_(fold change)| > 1. Expression levels were visualized in the form of a heatmap generated using R.

## 3. Results

### 3.1. Genome-Wide Identification of the PP2C Gene Family in Four Cotton Species

To identify PP2C family members in cotton, a comprehensive analysis was conducted using the sequences of 76 *Arabidopsis* PP2C proteins (TAIR, http://www.arabidopsis.org/ (accessed on 2 September 2025)), which were classified into ten subfamilies (A–J) and six singles (Table S1). These protein sequences were used as queries for local BLASTP and BLASTN searches against the protein and genomic databases of *G. barbadense* (AD_2_), *G. hirsutum* (AD_1_), *G. arboreum* (A_2_), and *G. raimondii* (D_5_). A stringent E-value cutoff of <1 × 10^−5^ was applied to generate an initial set of candidate PP2C genes. For further validation, the hidden Markov models (HMMs) of the PP2C protein domain (Pfam: PF00481 and PF07228) were downloaded from the Pfam database (http://pfam.xfam.org/ (accessed on 4 September 2025)), and used to scan the predicted protein datasets of the four cotton species via the HMMER software package. Candidates were retained only if they contained a complete PP2C domain with a length exceeding 100 amino acids and an E-value < 1 × 10^−5^. This rigorous process finally identified 105, 105, 204, and 152 putative PP2C genes in *G. arboreum*, *G. raimondii*, *G. hirsutum*, and *G. barbadense*, respectively (Table S2).

### 3.2. Chromosomal Distribution of PP2C Genes in Four Cotton Genomes

**Figure 1:**
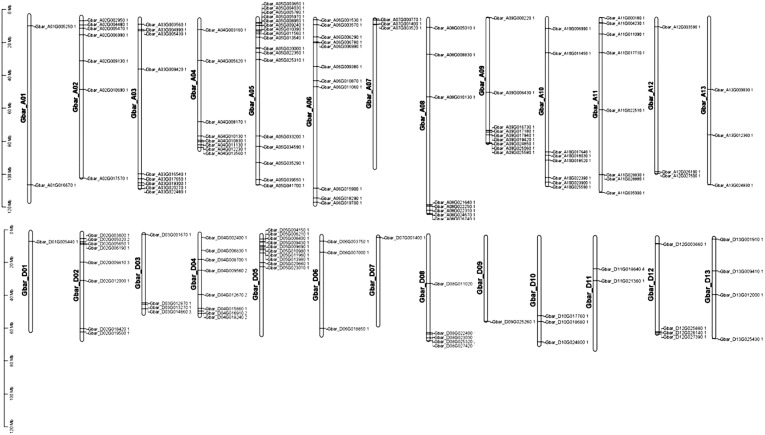
Chromosomal distribution of PP2C genes in *G. barbadense*. The map was generated using TBtools [19].

Chromosomal location analysis revealed that the 152 identified *G. barbadense* PP2C genes were unevenly distributed across all 26 chromosomes. The highest gene densities were observed on chromosomes A05 and D05, harboring 17 and 10 genes, respectively (Figure 1). Similarly, in *G. hirsutum*, all 204 PP2C genes were mapped to 26 chromosomes, with a notable abundance on chromosomes A05 and D05, involving 16 and 12 genes, respectively; the presence of numerous gene clusters in the *G. hirsutum* genome suggests that tandem duplication events have played a significant role in the expansion of the PP2C family in this species (Figure S1). In the diploid progenitors, the 105 *G. arboreum* (A_2_) PP2C genes were also predominantly located on chromosome Chr05, which contained 13 genes (Figure S2). Likewise, in *G. raimondii* (D_5_), the 105 PP2C genes were distributed across the chromosomes with the highest concentrations on chromosomes Chr05 that contained 13 genes (Figure S3). The strikingly similar distribution patterns between tetraploids *G. barbadense*, *G. hirsutum* and their diploid ancestors are likely attributable to evolutionary conservation, yet with divergent gains and losses in At and Dt subgenomes of *G. barbadense* and *G. hirsutum*.

### 3.3. Gene Structure and Conserved Motif Analysis of PP2Cs in Four Cotton Species

**Figure 2:**
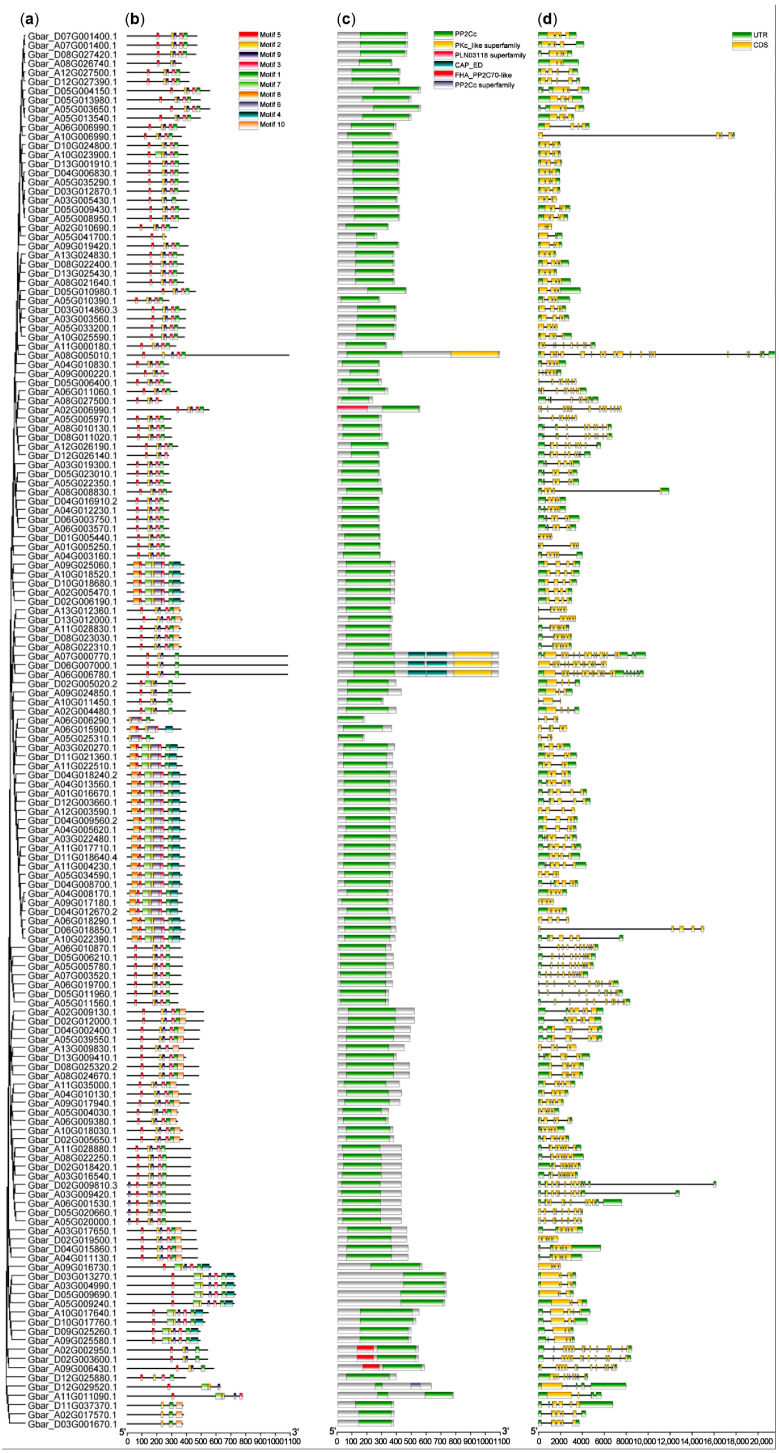
Gene structure and motif composition of PP2Cs in *G. barbadense*. (**a**) Phylogenetic grouping. The tree was constructed using MEGA 11 [21]. (**b**) Conserved motifs. On the right side, the top group of icons represent conserved motifs (motif 1–10) predicted by the MEME website. (**c**) PP2C domains. The middle group of icons denote six conserved PP2C protein related domains annotated using NCBI-CDD website. (**d**) Gene structure. The last two icons at the bottom represent gene structures, UTR and CDS. All above were visualized using TBtools [19].

To investigate the structural conservation and divergence of cotton PP2C gene family during evolution, we analyzed the gene structures and conserved protein motifs of the identified members in *G. barbadense* (AD_2_), *G. hirsutum* (AD1), *G. arboreum* (A_2_), and *G. raimondii* (D_5_). Phylogenetic trees were constructed based on coding sequence (CDS) and DNA sequences, and the corresponding gene structure and motif composition diagrams were generated (Figure 2 and Figures S4–S6).

Comparative analysis of exon-intron structures revealed significant diversity among PP2C genes. While most *G. arboreum* PP2C genes lacked untranslated regions (UTR) (Figure S5), genes in the other three species often contained UTR at both ends of the sequences (Figure 2, Figures S4 and S6). Furthermore, the number of exons varied considerably among different PP2C genes.

Conserved motif prediction using MEME version 5.5.8 software identified ten distinct motifs. Motif 1 was present in all four species and was found to overlap with the critical PP2Cc domain (PF00481), the primary functional region for phosphatase activity. The core sequence of this motif was identified as LTPEDEFLILASDGLWDVLSNZEAVDIVR (located between approximately 146–366 bp in the alignment). A combination of motifs 1, 2, 5, and 6, predominantly located in the central part of the proteins, forms a conserved domain of approximately 60 amino acid residues characteristic of the PP2C family. Notably, motif 5 was found in 98% and 96.6% of PP2C proteins in *G. barbadense* and *G. hirsutum*, respectively. Similarly, Motif 6 was present in 97% of *G. barbadense* PP2Cs and 100% of *G. hirsutum* PP2Cs. The high conservation and specific combination of these motifs suggest they are essential for the specific protein dephosphorylation functions of this gene family.

### 3.4. Phylogenetic Analysis of PP2C Genes in Gossypium

**Figure 3:**
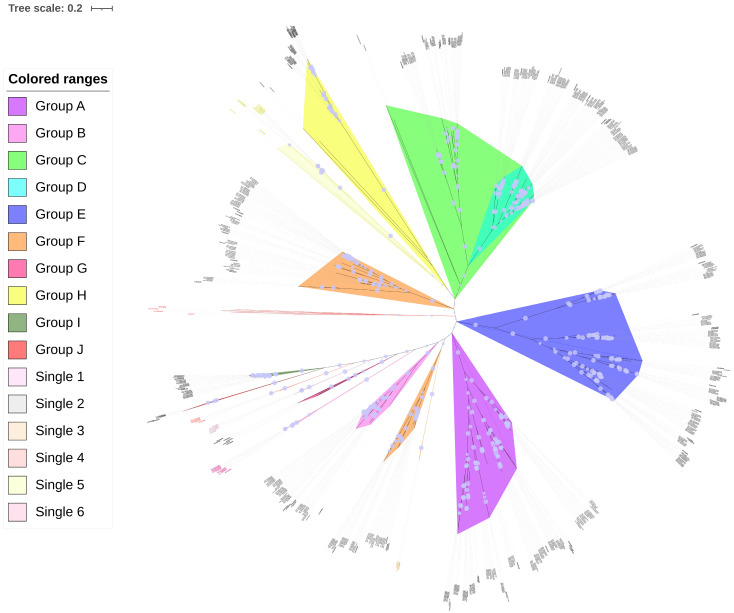
Phylogenetic tree of PP2C proteins from *G*. *barbadense* and *Arabidopsis*. Bootstrap support values from 1000 replicates are shown by light blue dots on the branches. The larger the dot, the higher the bootstrap value. The tree was built and visualized using MEGA 11 and iTOL software.

To investigate the evolutionary relationships of the PP2C gene family in *Gossypium*, a phylogenetic analysis was performed using the protein sequences of *Arabidopsis* PP2C proteins as an outgroup. Multiple sequence alignment was conducted with ClustalW, and the resulting alignment was used to construct a phylogenetic tree via the Maximum Likelihood (ML) method in MEGA 11.0 software. The Poisson correction was applied, and branch support was assessed with 1000 bootstrap replicates. Phylogenetic analysis grouped PP2C genes from *G. barbadense* (152), three other cotton species (*G. hirsutum*, 204; *G. arboreum*, 105; *G. raimondii*, 105), and *Arabidopsis* (76) into ten major clades (A–J) and six single clusters (Figure 3), indicating evolutionary conservation with cotton species and across different plant species. Most clades were monophyletic, except that clade F was divided into two distinct sections. Notably, Single 2 (containing AT1g75010) formed an outmost branch, while Single 3 (containing AT2g40860) was closely related to clade F, and Single 5 (containing AT4g11040) clustered near clade H. These clustering features suggest that the conventional 10-subfamily classification may warrant refinement in cotton, potentially expanding to up to 15 groups (Arranged clockwise in the circle diagram as: Single 2, F1, H + Single 5, C, D, E, A, F2 + Single 3, B, Single 1, G, Single 6, Single 4, J, I). Additionally, Single 1 (with AT5g19280) was positioned between clades B and G, whereas Singles 4 (AT4g27800) and 6 (AT1g18030) lay between clades G and J. Given their close phylogenetic relationships, these smaller groups (Single 1, G, Single 6, Single 4, J) could be consolidated, leading to a reduction in the total group number.

### 3.5. Physicochemical Properties and Subcellular Localization of PP2Cs in G. barbadense

**Figure 4:**
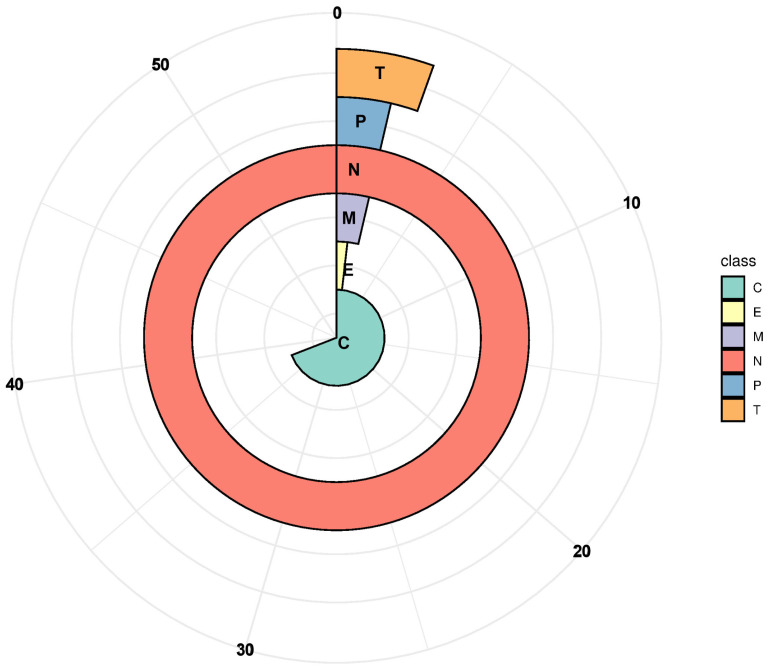
Subcellular localization of PP2Cs in *G. barbadense*. N, nucleus; C, chloroplast; M, mitochondria; T, cytosol; E, endoplasmic reticulum; P, plastid membrane. Subcellular localizations were predicted and visualized using TargetP-2.0 and R-4.5.2 softwares.

The physicochemical properties of the 152 identified *G. barbadense* PP2C proteins were analyzed using the ExPASy online tool. The protein lengths ranged from 179 to 1091 amino acids, with predicted molecular weights varying between 20.30 and 121.45 kDa and theoretical isoelectric points ranging from 4.53 to 9.36 (Table S3). Subcellular localization predictions performed with WoLF PSORT indicated that the majority of GbPP2C proteins are predominantly localized to the nucleus and chloroplasts (Figure 4, Table S3).

### 3.6. Protein Interaction Networks and Functional Enrichment of GbPP2Cs

To explore the functional associations of the PP2C proteins in *G. barbadense*, we predicted protein–protein interaction (PPI) networks and performed Gene Ontology (GO) and Kyoto Encyclopedia of Genes and Genomes (KEGG) enrichment analyses. Based on homology with *G. hirsutum* PP2C proteins, only one interactive pair was identified, namely, LOC107901268 (red, Gbar_A06G006780) and LOC107930842 (green, Gbar_A07G000770) (Figure S7). In contrast, analysis using *A. thaliana* homologs reveled three distinct networks associated with specific functional pathways: protein dephosphorylation (red, 18 PP2Cs), peptidyl-threonine dephosphorylation (green, 6 PP2Cs), and small network composed of “F17I5.110 and PAPP2C” (blue, 2 PP2Cs) (Figure S8). This discrepancy highlights the need for further experimental validation to elucidate the functional protein interactions of PP2Cs in cotton.

**Figure 5:**
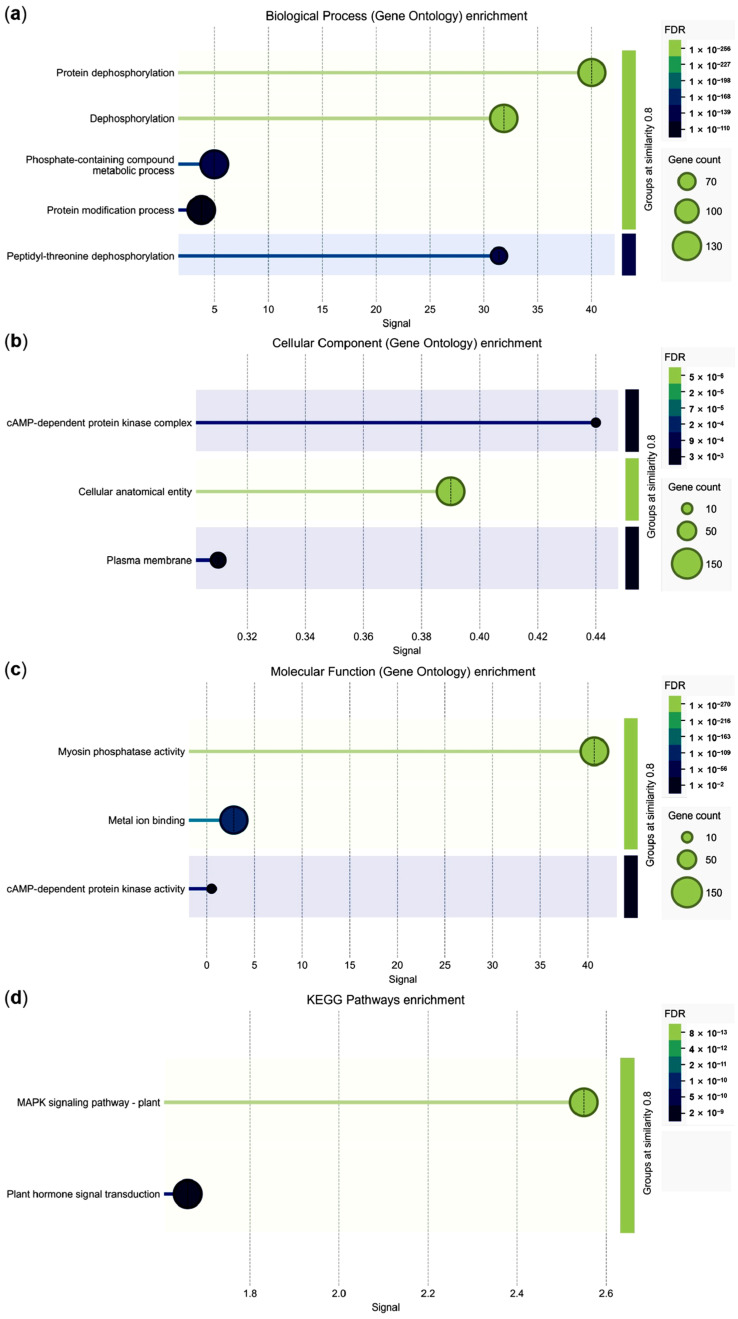
Functional enrichment analysis of GbPP2C proteins. (**a**) Biological process GO terms. (**b**) Cellular component GO terms. (**c**) Molecular function GO terms. (**d**) KEGG pathway enrichment. False discovery rate (FDR) < 0.05. The x-axis “Signal” represents the weighted harmonic mean of -log(FDR) and the observed/expected ratio. Signal > 0.01. GO and KEGG enrichment were analyzed and visualized using STRING software.

Functional enrichment analysis demonstrated that GbPP2Cs were most strongly associated with protein dephosphorylation in the biological process category (Figure 5a). For cellular components, they were significantly enriched in the cAMP-dependent protein kinase complex (Figure 5b). Molecular function analysis indicated that most GbPP2Cs possess myosin phosphatase activity (Figure 5c). Notably, a subset of 15 GbPP2C members showed concurrent enrichment in both the plant MAPK signaling pathway and plant hormone signal transduction, with particularly strong signals in the former (Figure 5d; Table S4). These findings provide valuable insights for further functional characterization of GbPP2Cs in these key signaling pathways.

### 3.7. Screening of GbPP2C Genes Related to Stress Resistance

Given the documented roles of PP2C members in plant disease resistance, we investigated which GbPP2Cs are associated with disease responses in *G. barbadense*. To identify genes involved in fungal resistance, we used RNA-seq data from hypocotyl tissues of *G. hirsutum* accession J668 following inoculation with *Fusarium oxysporum* f. sp. *vasinfectum* (*Fov*) [22]. Knocking out *Fov7* gene resulted in *G. hirsutum* lines (including KO#5) extremely susceptible to *Fov* race 7 [22], suggesting that *Fov7* is a positive regulator of *Fusarium wilt* (*FW*) resistance in cotton. Comparison between the susceptible *Fov7*-knockout line *Fov7*_KO#5 and the resistant genotype J668 at 10 days post-inoculation revealed four homologous GbPP2C genes (*Gbar_A04G010130*, *Gbar_A09G017940*, *Gbar_A13G012360*, and *Gbar_D13G012000*) that were significantly down-regulated in the susceptible *Fov*-infected *Fov7*_KO#5 line (Table 1). Therefore, we speculated that these four GbPP2Cs are also potential positive regulators of *FW* resistance.

**Figure 6:**
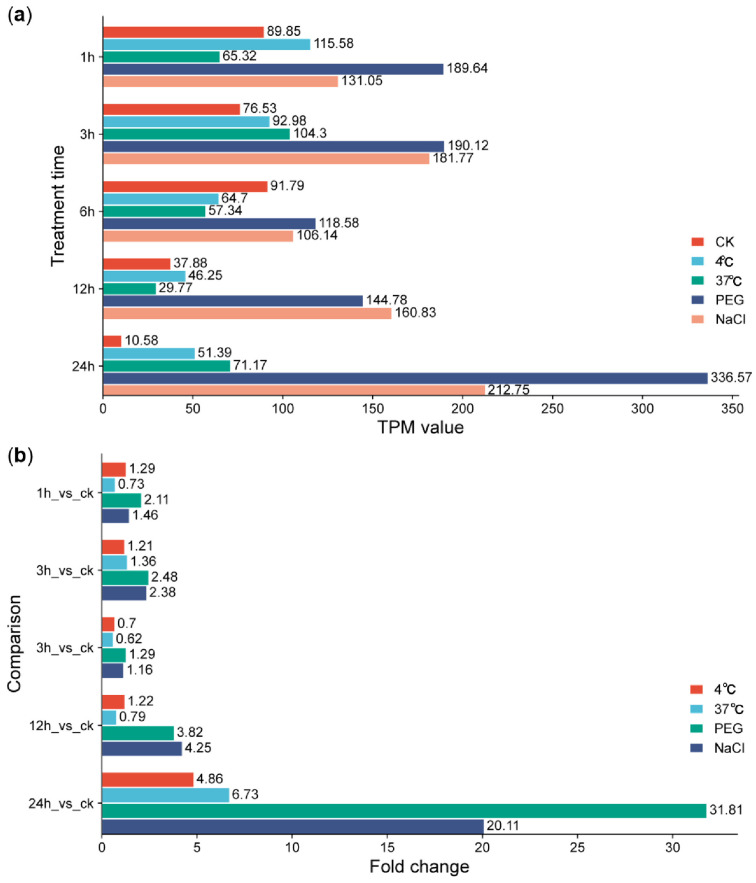
Expression level (**a**) and change fold (**b**) of *GbPP2C* gene *Gbar_D02G005650* after cold (4 °C), heat (37 °C), drought (PEG), and salt (NaCl) treatments in *G. barbadense* accession H7124. Expression levels were visualized using R software.

We further explored the involvement of GbPP2Cs in abiotic stress responses using expression data from G. barbadense accession H7124 subjected to cold, heat, drought, and salt treatments over a 24 h time course (0, 1, 3, 6, 12, 24 h) [23]. Phylogenetic analysis identified three distinct clades of GbPP2Cs preferentially induced by cold (48 genes), heat (12 genes), or both drought and salt (11 genes) (Figure S9a). Among these, *Gbar_D02G005650* emerged as a prominent candidate, showing markedly elevated expression levels under both drought (PEG) and salt (NaCl) stress, especially at 24 h (Figure S9b and Figure 6).

### 3.8. Identification of Candidate GbPP2Cs Associated with Fiber Development

*G. barbadense* is a major source of premium natural fibers due to its excellent fiber quality. Therefore, we sought to identify specific GbPP2C genes involved in fiber development. RNA-seq was performed on fibers at 0, 5, 10, 15, 20, and 25 days post-anthesis (DPA) from five *G. barbadense* accessions with contrasting fiber traits: XH37 (high strength), XH58 (long fiber), XH33 (low lint percentage), LuoSaiNa (low strength), and AShi (short fiber, high lint percentage) [1].

**Figure 7:**
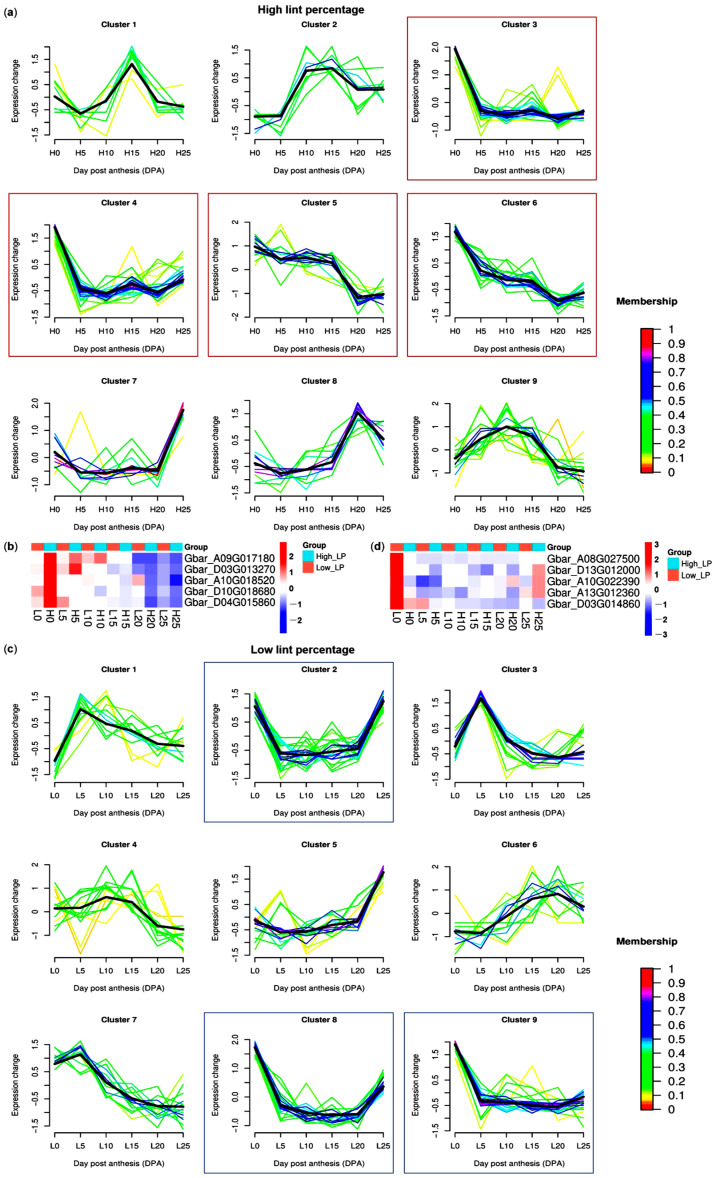
Identification of *GbPP2C* genes related to lint percentage (LP). (**a**) Mfuzz time series of *GbPP2C* genes during different fiber development stages of high-lint-percentage *G. barbadense* accession. Red boxes highlighted the candidates that positively regulated lint percentage. (**b**) Expression levels (z-scored FPKM values) of five potential LP-positive-regulator *GbPP2C*s during different fiber development stages between low- and high-lint-percentage *G. barbadense* accessions. (**c**) Mfuzz time series of *GbPP2C* genes during different fiber development stages of low-lint-percentage *G. barbadense* accession. Blue boxes highlighted the candidates that negatively regulated lint percentage. (**d**) Expression levels (z-scored FPKM values) of five potential LP-negative-regulator *GbPP2C*s during different fiber development stages between low- and high-lint-percentage *G. barbadense* accessions. Mfuzz time series and expression heatmaps were visualized using R software.

Fiber initiation stage (especially 0 DPA) is vital for lint formation. Here, we first performed mfuzz time series analysis of *GbPP2C* genes during different fiber development stages of high-lint-percentage *G. barbadense* accession. 96 GbPP2Cs highly expressed in 0 DPA were retrieved (clusters 3–6; Figure 7a). Among these, five genes (*Gbar_A09G017180*, *Gbar_D03G013270*, *Gbar_A10G018520*, *Gbar_D10G018680*, and *Gbar_D04G015860*) showed the highest expression and a fold change more than 2 at 0 DPA of high-lint-percentage genotype than low one (Figure 7b). Conversely, we obtained another five genes (*Gbar_A08G027500*, *Gbar_D13G012000*, *Gbar_A10G022390*, *Gbar_A13G012360*, and *Gbar_D03G014860*) with highest expression and more than 2 fold-change expression difference at 0 DPA of low lint-percentage (LP) accession (clusters 2, 8, 9; Figure 7c,d), suggesting roles as potential negative regulators of lint percentage. Notably, two genes (*Gbar_D13G012000* and *Gbar_A13G012360*) were also identified as potential positive regulators of *FW* resistance, implying the negative correlation between yield and disease resistance.

A similar strategy was adopted to mine the candidate *GbPP2C* genes regulating fiber length and strength, with highest expression at 5–20 and 25 DPA stages, respectively. For fiber length (FL), ten GbPP2C genes (*Gbar_D13G025430*, *Gbar_A13G024830*, *Gbar_A09G019420*, *Gbar_A08G026740*, *Gbar_A07G001400*, *Gbar_D07G001400*, *Gbar_A11G004230*, *Gbar_D08G027420*, *Gbar_A06G001530*, and *Gbar_D05G020660*) exhibited peak expression at 5 DPA and were identified as potential positive regulators of fiber length (Figure S10a,e). In contrast, five potential negative regulators (*Gbar_A05G004030*, *Gbar_A09G000220*, *Gbar_A03G005430*, *Gbar_D03G012870*, and *Gbar_A05G005780*) showed maximal expression at 15 and 20 DPA (Figure S10b,f). Amongst, we found three potential FL positive regulators (*Gbar_A09G019420*, *Gbar_D07G001400*, and *Gbar_D08G027420*) and one potential FL negative regulators (*Gbar_D03G012870*) that were enriched in two pathway, MAPK signaling pathway-plant and plant hormone signal transduction (Table S4), implying these genes might regulate FL via tuning MAPK and hormone signals. Regarding fiber strength (FS), only one positive regulator, *Gbar_A03G005430* (annotated as *GbAIP1*, encoding an AKT1-interacting phosphatase 1), met the criteria (FPKM > 1, fold change > 2; Figure S10c,d,g). Intriguingly, *GbAIP1* was also identified as a potential negative regulator of fiber length, indicating a potential trade-off between fiber length and strength. This expression trend was consistent across accessions with extreme fiber phenotypes (Figure S10f,g).

### 3.9. GbPP2C59 Might Negatively Regulate Early Maturity of G. barbadense

Given the roles of PP2C genes in various aspects of plant growth, we sought to identify specific PP2C family members potentially involved in regulating maturity in *G. barbadense*. This investigation aims to provide valuable genetic resources for breeding early-maturing cotton cultivars, a trait of particular importance for production in regions like Xinjiang. To this end, we conducted RNA-seq analyses on flowers and shoot apical meristems (SAM) from two *G. barbadense* accessions exhibiting extreme maturity phenotypes: the extremely late-maturity (LM) accession 5476-И (growth period: 137 days) and the extremely early-maturity (EM) accession KkH-8660 (growth period: 121 days). Gene expression levels were quantified and normalized as FPKM (Fragments Per Kilobase of exon per Million mapped reads) values.

**Figure 8:**
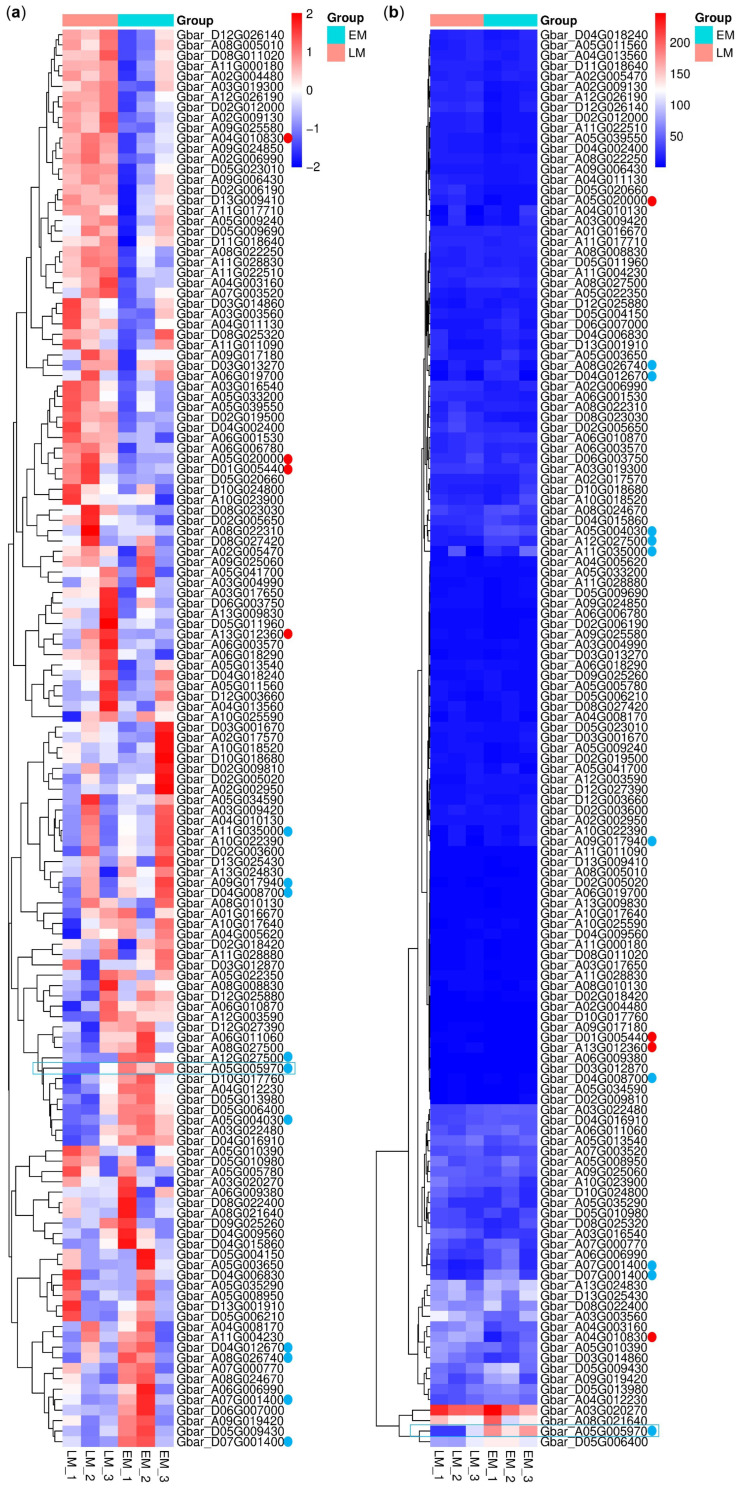
Expression profile of *GbPP2C* genes in LM and EM *G. barbadense* accessions. (**a**) Z-scored FPKM values. (**b**) Raw FPKM values. Dots, |log_2_(fold change)| > 1; blue, negative; red, positive. Blue box, the optimal negative candidate for maturity regulation of *G. barbadense*. Expression heatmaps were visualized using R software.

Among the 152 identified *G. barbadense* PP2C (*GbPP2C*) genes, 137 were reliably expressed (FPKM > 0) in the sampled tissues and thus retained for subsequent differential expression analysis. Comparative analysis between LM and EM accessions revealed 45 *GbPP2C* genes exhibiting higher expression in the LM accession (putative positive regulators of maturity), of which four genes showed a |log_2_(Fold Change)| > 1 with statistical significance (Figure 8, red dots). Conversely, 92 *GbPP2C* genes displayed higher expression in the EM accession (putative negative regulators of maturity), with ten of these genes exhibiting a |log_2_(Fold Change)| > 1 (Figure 8, blue dots).

Notably, the gene *Gbar_A05G005970*, annotated as encoding a probable protein phosphatase 2C 59 (named *GbPP2C59*), emerged as a particularly strong candidate. It displayed the most substantial difference (|log_2_(fold change)| = 1.89, *p* = 0.000195) among the differentially expressed *PP2C*s and was also characterized by relatively high expression abundance across the dataset (Figure 8, highlighted by blue box). Its significant upregulation in the early-maturity accession suggests a potential negative regulatory role in delaying maturation, making it a high-priority target for further functional validation studies aimed at manipulating maturity timing in *G. barbadense*.

## 4. Discussion

### 4.1. Expansion, Losses, and Conservatism of Cotton PP2C Gene Family During Evolution

Cotton is a classic example of an allotetraploid plant and serves as an excellent model for studying evolutionary analysis and polyploidization processes [24]. The PP2C gene family has been previously identified and characterized in several plant species, including *Arabidopsis* [5], rice [4], maize [25], Brachypodium [26], and banana [27]. While previous studies on the PP2C gene family in *Gossypium* species exist, our study employed distinct and more domain models, resulting in the identification of a larger repertoire of PP2C genes compared to prior report (147, 181, 87, and 99) [16]. In comparison, our study identified 152, 204, 105, and 105 PP2C genes in *G. barbadense* (AD_2_), *G. hirsutum* (AD_1_), *G. arboreum* (A_2_), and *G. raimondii* (D_5_) respectively. Given that the D_5_ and A_2_ genomes are considered putative donors of the allotetraploid cottons, the differences in gene numbers suggest that the PP2C gene family has undergone significant expansion from diploids to tetraploids. Furthermore, the variation in gene numbers between *G. barbadense*/*G. hirsutum* and the sum of diploid ancestors (*G. arboreum* and *G. raimondii*) implies that different degrees of gene losses have occurred during tetraploid formation and divergency. Additionally, our conserved motif analysis revealed that most PP2C proteins contain motifs 1, 5, and 6, confirming that all identified members exhibit typical family characteristics. Exon-intron organizations unveiled that PP2C genes within the same subgroup tend to exhibit similar gene structures. Our comprehensive phylogenetic analysis, which included PP2Cs from four cotton species and *Arabidopsis*, resolved the gene family into 15 distinct groups, thereby establishing a more detailed and robust classification system than was previously possible using only *G. hirsutum* and *Arabidopsis* sequences [16]. Protein functional enrichment analyses confirmed their shared role in protein dephosphorylation, together implying a broad conservation of core PP2C functions during the evolution of different plant species.

### 4.2. PP2C-Mediated Biotic and Abiotic Stress Adaptation in G. barbadense

The PP2C gene family, as the largest group of protein phosphatases in plants, serves as a key regulator of dephosphorylation events in various signaling pathways, including those involved in disease responses [9]. Previous studies have established their importance in plant immunity. In *Arabidopsis*, double mutants lacking *AtPP2C62* and *AtPP2C26* exhibited reduced disease symptoms and suppressed bacterial multiplication upon infection with *Xanthomonas oryzae* pv. *oryzae* [28]. Similarly, three *Arabidopsis* PP2Cs (HAI1, HAI2, and HAI3) were essential for ABA-mediated MPK3/MPK6 inactivation and immune suppression [10]. In *G. barbadense*, we previously identified a disease-resistance gene *GbPP2C80* that interacted with GbWAKL14 to negatively co-regulate resistance to *Fusarium* and *Verticillium wilt* through MPK3 and ROS signaling [3]. In upland cotton (*G. hirsutum*), a major-effect loci, *Fov7,* was identified as a positive *FW*-resistance regulator [22]. The present study further identified four GbPP2C genes as potential positive regulators of *FW* resistance, based on their significant down-regulation in the susceptible *Fov7*-knockout line compared to the resistant genotype J668 at 10 days post-inoculation. The shared GO annotation of these four genes in protein dephosphorylation suggests a potential mechanism whereby they contribute to disease resistance through the dephosphorylation of key signaling components, such as MPKs.

Beyond biotic stress, PP2Cs are crucial mediators of abiotic stress adaptation, such as drought, salt, cold, and hot, etc. In *G. hirsutum*, PP2C protein GhDRP1 has been reported to function as a negative regulator of drought tolerance through ABA and flavonoid metabolism [11]. Similarly, *GhHAI2*, *GhAHG3*, and *GhABI2* also negatively modulated osmotic stress via ABA pathway [29]. Furthermore, 30 GhPP2C genes were induced by different abiotic stresses, including heat, cold, drought, and salt [16]. However, the role of PP2Cs in abiotic stress adaptation in *G. barbadense* remained unexplored. Herein, our expression profiling of *G. barbadense* under cold, heat, drought, and salt treatments revealed 48, 12, and 11 GbPP2C genes preferentially induced by cold, heat, and both drought/salt stresses, respectively. Notably, *Gbar_D02G005650* exhibited consistently elevated expression under all four stress conditions, particularly under PEG and NaCl treatments, highlighting its potential as a pleiotropic regulator for multi-stress tolerance breeding. Given the established role of PP2C-mediated ABA signaling in abiotic stress responses, it is plausible that the identified candidate gene modulates abiotic stress tolerance in *G. barbadense* through a similar regulatory pathway.

### 4.3. Roles of PP2Cs in Fiber Morphogenesis and Growth Regulation of G. barbadense

Although previous transcriptomic analyses in upland cotton revealed expression patterns of PP2Cs during fiber development [16], specific regulators of fiber traits remained unidentified. Our systematic screening using RNA-seq data from multiple developmental stages of *G. barbadense* accessions with extreme fiber performance identified potential GbPP2C regulators for lint percentage, fiber length and strength [1]. We discovered five potential positive and five potential negative GbPP2C regulators of lint percentage, among which two negative ones (*Gbar_D13G012000* and *Gbar_A13G012360*) also function as potential positive regulators of *FW* resistance, suggesting their potential for balancing yield and disease resistance. For fiber length, ten GbPP2Cs were characterized as potential positive regulators and five as potential negative regulators. Notably, three positive (*Gbar_A09G019420*, *Gbar_D07G001400*, and *Gbar_D08G027420*) and one negative (*Gbar_D03G012870*) regulators of fiber length might function through MAPK and hormone signaling pathways. Similarity, silencing GhKRP6 (a cotton cell cycle-dependent kinase inhibitor) caused differential expression of genes related to cell wall biosynthesis, MAPK, and plant hormone transduction pathways—all of which are related to cell expansion [30]. Notably, *Gbar_A03G005430* (*GbAIP1*) was identified as a positive regulator of fiber strength but a negative regulator of fiber length, indicating its role as a key hub gene coordinating these two critical quality attributes. Currently, no direct evidence links AIP1 genes to cotton fiber development. The sole functional report on AIP1 indicates its role in regulating ammonium transport by dephosphorylating AMMONIUM TRANSPORTER 1 (AMT1) proteins [31]. Expanding to the broader context of protein phosphatases in fiber development, previous studies show that Protein Phosphatase 2A (PP2A) influences fiber length by modulating end-binding protein 1C (GhEB1C) to regulate microtubule dynamics [32]. Additionally, Leafy Cotyledon1-Like 1 (GhL1L1) activates PHOSPHATASE 2AA2 (GhPP2AA2), which subsequently regulates GhPIN1 activity to determine cell fate specification [33]. Based on these findings, we hypothesize that AIP1 may influence fiber development through potential roles in ammonium transport, microtubule dynamics, and auxin distribution.

Beyond their roles in stress responses, PP2Cs actively participate in regulating various aspects of plant growth and development [9]. For instance, silencing of SlPP2C in tomato significantly delayed senescence and ripening in leaves, flowers, and fruits; although inducible by ethylene (ETH) and ABA, SlPP2C did not appear to interact directly with ABA receptors [14]. In *Brassica juncea*, BjuPP2C52 expression increased during reproductive growth stages, where it interacted with photoperiod regulator BjuFKF1 and seven other proteins to fine-tune flowering time [15]. Similarly, in upland cotton, higher expression of ABA-related PP2Cs genes correlated with lower plant height, and higher auxin and ABA levels, in a dwarf mutant of *G. hirsutum* [34]. Furthermore, *GhPP2C1-5* genes may contribute to dwarfing regulated by *Ammopiptanthus mongolicus* C-repeat binding factor 1 (*AmCBF1)*, and silencing of *GhPP2C1*/*2* in R15 impaired cotton growth [35]. Despite these documented roles, the function of PP2Cs in regulating growth period in *G. barbadense* remained unexplored. Our comparison of extreme early- and late-maturity *G. barbadense* accessions identified *Gbar_A05G005970* (*GbPP2C59*), which exhibited higher expression in early-maturity accession, suggesting it acted as a negative regulator of growth period. We hypothesize that GbPP2C59 may promote early maturation by modulating key signaling pathways, potentially through dephosphorylation of kinases in the ABA signaling cascade to release growth inhibition, and/or by regulating components of the photoperiod pathways to accelerate the vegetative-to-reproductive transition.

While our in silico analyses provide comprehensive insights into the PP2C gene family in G. barbadense, it is important to note that these predictions require experimental validation. Future work should include functional studies such as gene knockout/knockdown experiments, protein–protein interaction assays, and detailed phenotypic characterization under stress conditions to confirm the roles of the candidate genes identified in this study.

## 5. Conclusions

This study presents a comprehensive genome-wide analysis of the PP2C gene family in *G. barbadense*, identifying 152 members that exhibit remarkable evolutionary conservation and lineage-specific expansion. Through systematic phylogenetic, structural, interactive network, functional enrichment, and expression analyses, we have elucidated the functional diversification of GbPP2Cs in regulating key agronomic traits. We identified multiple GbPP2Cs responsive to both biotic and abiotic stresses, including four genes associated with *FW* resistance and one gene (*Gbar_D02G005650*) showing induction under drought, salt, and temperature stresses at the same time. Furthermore, our findings demonstrate that specific GbPP2Cs showed preferential expression during critical stages of lint initiation, fiber elongation, and secondary cell wall thickening. Particularly noteworthy is the discovery of pleiotropic regulators such as *Gbar_A03G005430* (*GbAIP1*), which appears to coordinate the trade-off between fiber length and strength, and *Gbar_D13G012000* and *Gbar_A13G012360*, which may simultaneously regulate lint percentage and disease resistance. Additionally, we also identified *GbPP2C59* as a potential negative regulator of maturity in *G. barbadense*. These findings substantially expand our understanding of the PP2C family in cotton and provide valuable genetic resources for molecular breeding programs aimed at developing cotton varieties with enhanced stress resilience, superior fiber quality, and earlier maturity. Future research should focus on functional validation using approaches such as CRISPR-Cas9 mediated gene editing, VIGS (virus-induced gene silencing), and transgenic overexpression in model plants and cotton. For breeding applications, the pleiotropic regulators identified in this study offer exciting possibilities, for instance, developing tissue-specific or inducible promoters to fine-tune GbAIP1 expression for optimal fiber quality, or pyramiding positive resistance regulators like Gbar_D13G012000 through marker-assisted selection to enhance multi-stress resilience without compromising yield.

## Figures and Tables

**Table 1:** Downregulated *GbPP2C*s in hypocotyls of *Fov*-infected Fov7_KO#5 versus J668 at 10 dpi.

GbPP2C Gene	log_2_(Fold Change)	*p* Value	GO Term
*Gbar_A04G010130*	−3.040156774	8.33 × 10^−4^	GO:0006470 protein dephosphorylation
*Gbar_A09G017940*	−2.431645884	1.64 × 10^−4^	
*Gbar_A13G012360*	−2.423987924	6.70 × 10^−20^	
*Gbar_D13G012000*	−2.219528153	1.96 × 10^−3^	

## Data Availability

The original contributions presented in this study are included in the article/Supplementary Material. The raw data of fiber transcriptome sequencing can be obtained on the NCBI Gene Expression Omnibus (GEO) website, with the accession number GSE184965. Further inquiries can be directed to the authors.

## Supplementary Materials

The following supporting information can be downloaded at: https://www.mdpi.com/article/10.3390/cimb47120977/s1, Table S1: Information of *Arabidopsis* PP2Cs; Table S2: PP2C family members in four cotton species; Figure S1: Chromosomal distribution of PP2C genes in *G. hirsutum*; Figure S2: Chromosomal distribution of PP2C genes in *G. arboreum*; Figure S3: Chromosomal distribution of PP2C genes in *G. raimondii*; Figure S4: Gene structure and motif composition of PP2Cs in *G. hirsutum*; Figure S5: Gene structure and motif composition of PP2Cs in *G. arboreum*; Figure S6: Gene structure and motif composition of PP2Cs in *G. raimondii*; Table S3: Characteristics of PP2C genes in *G. barbadense*; Figure S7: Predicted protein–protein interactive networks of GbPP2Cs according to their homologous proteins in *G. hirsutum;* Figure S8: Predicted protein–protein interactive networks of GbPP2Cs according to their homologous proteins in *A. thaliana*; Table S4: KEGG enrichment of GbPP2Cs; Figure S9: Expression level of GbPP2C genes after cold (4 °C), heat (37 °C), drought (PEG), and salt (NaCl) treatments in *G. barbadense* accession H7124; Figure S10: Identification of GbPP2C genes related to fiber quality.
