# Supplementary material for: Genome-Wide Identification and Characterization of the PP2C Gene Family in Gossypium barbadense Reveals Potential Candidates for Breeding Improved Stress Resistance, Fiber Character, and Early Maturing Cotton Varieties
Source: Curr Issues Mol Biol. 2025 Nov 24;47(12):977. doi: 10.3390/cimb47120977 (PMC12731475; doi:10.3390/cimb47120977)
Supplement: Supplementary file 1 [file cimb-47-00977-s001.zip › cimb-3963160-supplementary.pdf]

current issues in molecular biology

MDPI

Article

Genome-Wide Identification and Characterization of the PP2C Gene Family in *Gossypium barbadense* Reveals Potential Candidates for Breeding Improved Stress Resistance, Fiber Character, and Early Maturing Cotton Varieties

Nan Zhao [1,†], Weiran Wang [1,†], Zixin Zhou [1], Meng Wang [1], Caixia Li [1], Lingfang Ran [1], Yaohua Li [1], Jianping Li [1], Jiahui Zhu [1], Zhiqing Liu [1], Yifan Wang [1], Yahui Deng [1], Jing Yang [1], Alifu Aierxi [1,*] and Jie Kong [1,*]

Supplementary Materials

Table S1. Information of *Arabidopsis* PP2Cs.

No.	Genomic_Locus	Sub_Family	Gene_Name	Protein_Function
1	AT4g26080	Group A	ABI1	protein phosphatase 2C ABI1
2	AT5g57050	Group A	ABI2	protein phosphatase 2C ABI2
3	AT1g72770	Group A	HAB1	protein phosphatase 2C P2C-HA
4	AT1g07430	Group A	NULL	protein phosphatase 2C, putative
5	AT2g29380	Group A	NULL	protein phosphatase 2C, putative
6	AT3g11410	Group A	AtPP2CA	protein phosphatase 2C, putative
7	AT5g51760	Group A	NULL	protein phosphatase 2C, putative
8	AT5g59220	Group A	NULL	protein phosphatase 2C, putative
9	AT1g17550	Group A	HAB2	protein phosphatase 2C-related
10	AT1g07160	Group B	AP2C2	protein phosphatase 2C, putative
11	AT1g67820	Group B	AP2C4	protein phosphatase 2C, putative
12	AT2g30020	Group B	AP2C1	protein phosphatase 2C, putative
13	AT2g40180	Group B	AthPP2C5/AP2C3	protein phosphatase 2C, putative
14	AT3g27140	Group B	AP2C6	protein phosphatase 2C, putative
15	AT4g08260	Group B	AP2C5	protein phosphatase 2C, putative
16	AT2g28890	Group C	AtPLL4	protein phosphatase 2C family protein
17	AT2g35350	Group C	AtPLL1	protein phosphatase 2C family protein
18	AT3g09400	Group C	AtPLL3	protein phosphatase 2C family protein
19	AT5g02400	Group C	AtPLL2	protein phosphatase 2C family protein
20	AT1g07630	Group C	AtPLL5	protein phosphatase 2C family protein
21	AT2g46920	Group C	AtPOL	protein phosphatase 2C family protein
22	AT3g16560	Group C	NULL	protein phosphatase 2C-related
23	AT3g12620	Group D	NULL	protein phosphatase 2C family protein
24	AT4g33920	Group D	NULL	protein phosphatase 2C family protein
25	AT4g38520	Group D	NULL	protein phosphatase 2C family protein
26	AT5g02760	Group D	NULL	protein phosphatase 2C family protein
27	AT5g66080	Group D	NULL	protein phosphatase 2C family protein

 https://doi.org/10.3390/cimb47120977

28	AT3g17090	Group D	NULL	protein phosphatase 2C family protein
29	AT5g06750	Group D	NULL	protein phosphatase 2C family protein
30	AT3g51370	Group D	NULL	protein phosphatase 2C, putative
31	AT3g55050	Group D	NULL	serine/threonine protein phosphatase 2C (PP2C6)
32	AT1g03590	Group E	AtPP2C6-6	protein phosphatase 2C family protein
33	AT1g16220	Group E	NULL	protein phosphatase 2C family protein
34	AT1g79630	Group E	NULL	protein phosphatase 2C family protein
35	AT3g02750	Group E	NULL	protein phosphatase 2C family protein
36	AT2g20050	Group E	NULL	protein phosphatase 2C, putative
37	AT3g05640	Group E	NULL	protein phosphatase 2C, putative
38	AT3g06270	Group E	NULL	protein phosphatase 2C, putative
39	AT3g16800	Group E	NULL	protein phosphatase 2C, putative
40	AT4g32950	Group E	NULL	protein phosphatase 2C, putative
41	AT5g01700	Group E	NULL	protein phosphatase 2C, putative
42	AT5g26010	Group E	NULL	protein phosphatase 2C, putative
43	AT5g36250	Group E	NULL	protein phosphatase 2C, putative
44	AT5g27930	Group E	AtPP2C6-7	protein phosphatase 2C, putative
45	AT1g34750	Group F	NULL	protein phosphatase 2C, putative
46	AT1g43900	Group F	NULL	protein phosphatase 2C, putative
47	AT2g34740	Group F	NULL	protein phosphatase 2C, putative
48	AT3g15260	Group F	NULL	protein phosphatase 2C, putative
49	AT3g23360	Group F	NULL	protein phosphatase 2C, putative
50	AT4g31750	Group F	NULL	protein phosphatase 2C, putative
51	AT5g24940	Group F	NULL	protein phosphatase 2C, putative
52	AT1g22280	Group F	NULL	protein phosphatase 2C, putative
53	AT1g78200	Group F	NULL	protein phosphatase 2C, putative
54	AT2g20630	Group F	NULL	protein phosphatase 2C, putative
55	AT4g28400	Group F	NULL	protein phosphatase 2C, putative
56	AT5g53140	Group F	NULL	protein phosphatase 2C, putative
57	AT5g10740	Group F	NULL	protein phosphatase 2C-related
58	AT2g25620	Group G	NULL	protein phosphatase 2C, putative
59	AT2g33700	Group G	NULL	protein phosphatase 2C, putative
60	AT3g51470	Group G	NULL	protein phosphatase 2C, putative
61	AT3g62260	Group G	NULL	protein phosphatase 2C, putative
62	AT1g48040	Group G	NULL	protein phosphatase 2C-related
63	AT3g17250	Group G	NULL	protein phosphatase 2C-related
64	AT1g09160	Group H	NULL	protein phosphatase 2C-related
65	AT1g68410	Group H	NULL	protein phosphatase 2C-related
66	AT1g47380	Group H	NULL	protein phosphatase 2C-related
67	AT2g25070	Group I	NULL	protein phosphatase 2C, putative
68	AT4g31860	Group I	NULL	protein phosphatase 2C, putative

69	AT3g63320	Group J	NULL	protein phosphatase 2C-related
70	AT3g63340	Group J	NULL	protein phosphatase 2C-related
71	AT5g19280	Single 1	KAPP	kinase associated protein phosphatase (KAPP)
72	AT1g75010	Single 2	NULL	MORN (Membrane Occupation and Recognition Nexus) repeat-containing protein
73	AT2g40860	Single 3	NULL	protein phosphatase 2C family protein
74	AT4g27800	Single 4	PPH1	protein phosphatase 2C PPH1
75	AT4g11040	Single 5	NULL	protein phosphatase 2C, putative
76	AT1g18030	Single 6	NULL	protein phosphatase 2C, putative

Table S2. PP2C family members in four cotton species.

No.	A_2_GaPP2Cs	D_5_GoPP2Cs	AD_1_GhPP2Cs	AD_2_GbPP2Cs
1	Ga01G0640	Gorai.001G007600	GhirA01G005260	Gbar_A01G005250
2	Ga01G1753	Gorai.001G013500	GhirA01G016260	Gbar_A01G016670
3	Ga01G2218	Gorai.001G038400	GhirA02G003130	Gbar_A02G002950
4	Ga01G2268	Gorai.002G068200	GhirA02G004590	Gbar_A02G004480
5	Ga01G2526	Gorai.002G199700	GhirA02G005190	Gbar_A02G005470
6	Ga02G0165	Gorai.003G016400	GhirA02G005590	Gbar_A02G006990
7	Ga02G1135	Gorai.003G080700	GhirA02G007170	Gbar_A02G009130
8	Ga03G0355	Gorai.003G128900	GhirA02G009420	Gbar_A02G010690
9	Ga03G0611	Gorai.003G132200	GhirA02G010880	Gbar_A02G017570
10	Ga03G0613	Gorai.003G146400	GhirA02G017990	Gbar_A03G003560
11	Ga03G0807	Gorai.004G056100	GhirA03G003580	Gbar_A03G004990
12	Ga03G0998	Gorai.004G097000	GhirA03G004990	Gbar_A03G005430
13	Ga03G1992	Gorai.004G115400	GhirA03G005380	Gbar_A03G009420
14	Ga03G2109	Gorai.004G232300	GhirA03G009410	Gbar_A03G016540
15	Ga03G2282	Gorai.004G237900	GhirA03G016510	Gbar_A03G017650
16	Ga03G2304	Gorai.004G238400	GhirA03G017590	Gbar_A03G019300
17	Ga03G2409	Gorai.004G263300	GhirA03G019020	Gbar_A03G020270
18	Ga03G2660	Gorai.004G284400	GhirA03G019240	Gbar_A03G022480
19	Ga04G0189	Gorai.004G293300	GhirA03G020190	Gbar_A04G003160
20	Ga04G0248	Gorai.005G041400	GhirA03G022370	Gbar_A04G005620
21	Ga04G0453	Gorai.005G055900	GhirA04G003480	Gbar_A04G008170
22	Ga04G0484	Gorai.005G062200	GhirA04G006030	Gbar_A04G010130
23	Ga04G0568	Gorai.005G067600	GhirA04G008560	Gbar_A04G010830
24	Ga04G0784	Gorai.005G084400	GhirA04G010370	Gbar_A04G011130
25	Ga04G1095	Gorai.005G103200	GhirA04G011040	Gbar_A04G012230
26	Ga04G1159	Gorai.005G126900	GhirA04G011300	Gbar_A04G013560
27	Ga04G1336	Gorai.005G192600	GhirA04G012320	Gbar_A05G003650
28	Ga04G1386	Gorai.005G204100	GhirA04G013690	Gbar_A05G004030
29	Ga04G1872	Gorai.005G218800	GhirA05G004200	Gbar_A05G005780
30	Ga04G2111	Gorai.005G221200	GhirA05G004590	Gbar_A05G005970

31	Ga05G0430	Gorai.005G233000	GhirA05G006240	Gbar_A05G008950
32	Ga05G0470	Gorai.005G255000	GhirA05G006440	Gbar_A05G009240
33	Ga05G0642	Gorai.006G001800	GhirA05G009480	Gbar_A05G010390
34	Ga05G0662	Gorai.006G053000	GhirA05G009790	Gbar_A05G011560
35	Ga05G0980	Gorai.006G172900	GhirA05G010990	Gbar_A05G013540
36	Ga05G1012	Gorai.006G177400	GhirA05G012160	Gbar_A05G020000
37	Ga05G1139	Gorai.006G185900	GhirA05G014140	Gbar_A05G022350
38	Ga05G1248	Gorai.006G201000	GhirA05G020690	Gbar_A05G025310
39	Ga05G1455	Gorai.006G252800	GhirA05G023080	Gbar_A05G033200
40	Ga05G2140	Gorai.006G255900	GhirA05G034260	Gbar_A05G034590
41	Ga05G2400	Gorai.006G257900	GhirA05G035520	Gbar_A05G035290
42	Ga05G3842	Gorai.006G263300	GhirA05G036190	Gbar_A05G039550
43	Ga05G3845	Gorai.007G006600	GhirA05G040490	Gbar_A05G041700
44	Ga06G0190	Gorai.007G047600	GhirA05G042640	Gbar_A06G001530
45	Ga06G0347	Gorai.007G118600	GhirA06G001630	Gbar_A06G003570
46	Ga06G0650	Gorai.007G190900	GhirA06G003710	Gbar_A06G006290
47	Ga06G0678	Gorai.007G220800	GhirA06G006850	Gbar_A06G006780
48	Ga06G1011	Gorai.007G302900	GhirA06G007030	Gbar_A06G006990
49	Ga06G1186	Gorai.007G303500	GhirA06G009470	Gbar_A06G009380
50	Ga06G1218	Gorai.007G379200	GhirA06G010970	Gbar_A06G010870
51	Ga06G2227	Gorai.008G037200	GhirA06G011170	Gbar_A06G011060
52	Ga06G2246	Gorai.008G022800	GhirA06G018260	Gbar_A06G015900
53	Ga07G0046	Gorai.008G267600	GhirA06G019690	Gbar_A06G018290
54	Ga07G0142	Gorai.008G269800	GhirA07G000420	Gbar_A06G019700
55	Ga07G0392	Gorai.008G270000	GhirA07G001370	Gbar_A07G000770
56	Ga08G0532	Gorai.008G282600	GhirA07G003730	Gbar_A07G001400
57	Ga08G0965	Gorai.009G042600	GhirA08G005020	Gbar_A07G003520
58	Ga08G1169	Gorai.009G046600	GhirA08G008730	Gbar_A08G005010
59	Ga08G2356	Gorai.009G063600	GhirA08G010000	Gbar_A08G008830
60	Ga08G2421	Gorai.009G065500	GhirA08G021030	Gbar_A08G010130
61	Ga08G2427	Gorai.009G096200	GhirA08G021630	Gbar_A08G021640
62	Ga08G2675	Gorai.009G099400	GhirA08G021680	Gbar_A08G022250
63	Ga08G2885	Gorai.009G111500	GhirA08G023800	Gbar_A08G022310
64	Ga08G2969	Gorai.009G122700	GhirA08G025840	Gbar_A08G024670
65	Ga09G0020	Gorai.009G143300	GhirA09G000170	Gbar_A08G026740
66	Ga09G0758	Gorai.009G211100	GhirA09G004220	Gbar_A08G027500
67	Ga09G1769	Gorai.009G236500	GhirA09G016460	Gbar_A09G000220
68	Ga09G1814	Gorai.009G379300	GhirA09G016900	Gbar_A09G006430
69	Ga09G1831	Gorai.009G421600	GhirA09G017690	Gbar_A09G016730
70	Ga09G2061	Gorai.010G018200	GhirA09G019200	Gbar_A09G017180
71	Ga09G2614	Gorai.010G040100	GhirA09G024360	Gbar_A09G017940
72	Ga09G2645	Gorai.010G074600	GhirA09G024630	Gbar_A09G019420

73	Ga09G2666	Gorai.010G076700	GhirA09G024830	Gbar_A09G024850
74	Ga09G2722	Gorai.010G102900	GhirA09G025360	Gbar_A09G025060
75	Ga10G0089	Gorai.010G119300	GhirA10G006270	Gbar_A09G025580
76	Ga10G0262	Gorai.010G120600	GhirA10G009270	Gbar_A10G006990
77	Ga10G0328	Gorai.010G138600	GhirA10G016710	Gbar_A10G011450
78	Ga10G0517	Gorai.010G203500	GhirA10G016700	Gbar_A10G017640
79	Ga10G1010	Gorai.010G222200	GhirA10G017090	Gbar_A10G018030
80	Ga10G1059	Gorai.011G071200	GhirA10G017700	Gbar_A10G018520
81	Ga10G1145	Gorai.011G181400	GhirA10G021230	Gbar_A10G022390
82	Ga10G1219	Gorai.011G189700	GhirA10G022700	Gbar_A10G023900
83	Ga10G2392	Gorai.011G194000	GhirA10G024400	Gbar_A10G025590
84	Ga11G0012	Gorai.011G200800	GhirA11G000600	Gbar_A11G000180
85	Ga11G0823	Gorai.011G242300	GhirA11G004660	Gbar_A11G004230
86	Ga11G0828	Gorai.011G288200	GhirA11G011390	Gbar_A11G011090
87	Ga11G1848	Gorai.011G292300	GhirA11G018170	Gbar_A11G017710
88	Ga11G2186	Gorai.012G003400	GhirA11G023140	Gbar_A11G022510
89	Ga11G2938	Gorai.012G026700	GhirA11G029270	Gbar_A11G028830
90	Ga11G3648	Gorai.012G072200	GhirA11G029330	Gbar_A11G028880
91	Ga11G4068	Gorai.012G077700	GhirA11G035590	Gbar_A11G035000
92	Ga12G0156	Gorai.012G092200	GhirA12G003680	Gbar_A12G003590
93	Ga12G0285	Gorai.012G100800	GhirA12G022260	Gbar_A12G026190
94	Ga12G0287	Gorai.012G119300	GhirA12G026170	Gbar_A12G027500
95	Ga12G0309	Gorai.012G126900	GhirA12G026380	Gbar_A13G009830
96	Ga12G0700	Gorai.012G130800	GhirA12G026400	Gbar_A13G012360
97	Ga12G2685	Gorai.012G142300	GhirA12G027630	Gbar_A13G024830
98	Ga13G0011	Gorai.012G156600	GhirA13G001840	Gbar_D01G005440
99	Ga13G0220	Gorai.013G001000	GhirA13G009370	Gbar_D02G003600
100	Ga13G0440	Gorai.013G022100	GhirA13G011580	Gbar_D02G005020
101	Ga13G1038	Gorai.013G049000	GhirA13G020900	Gbar_D02G005650
102	Ga13G1195	Gorai.013G101600	GhirA13G024610	Gbar_D02G006190
103	Ga13G1395	Gorai.013G127100	GhirD01G005490	Gbar_D02G009810
104	Ga13G2432	Gorai.013G230300	GhirD01G017770	Gbar_D02G012000
105	Ga13G2819	Gorai.013G269500	GhirD02G003500	Gbar_D02G018420
106			GhirD02G004910	Gbar_D02G019500
107			GhirD02G005510	Gbar_D03G001670
108			GhirD02G005950	Gbar_D03G012870
109			GhirD02G007590	Gbar_D03G013270
110			GhirD02G009350	Gbar_D03G014860
111			GhirD02G011490	Gbar_D04G002400
112			GhirD02G017780	Gbar_D04G006830
113			GhirD02G018900	Gbar_D04G008700
114			GhirD02G020590	Gbar_D04G009560

115	GhirD02G021610	Gbar_D04G012670
116	GhirD02G023810	Gbar_D04G015860
117	GhirD03G001620	Gbar_D04G016910
118	GhirD03G009040	Gbar_D04G018240
119	GhirD03G013400	Gbar_D05G004150
120	GhirD03G013770	Gbar_D05G006210
121	GhirD03G015370	Gbar_D05G006400
122	GhirD04G000250	Gbar_D05G009430
123	GhirD04G002490	Gbar_D05G009690
124	GhirD04G006870	Gbar_D05G010980
125	GhirD04G007360	Gbar_D05G011960
126	GhirD04G008710	Gbar_D05G013980
127	GhirD04G009460	Gbar_D05G020660
128	GhirD04G012630	Gbar_D05G023010
129	GhirD04G014590	Gbar_D06G003750
130	GhirD04G015290	Gbar_D06G007000
131	GhirD04G015660	Gbar_D06G018850
132	GhirD04G016740	Gbar_D07G001400
133	GhirD04G018100	Gbar_D08G011020
134	GhirD05G000090	Gbar_D08G022400
135	GhirD05G004320	Gbar_D08G023030
136	GhirD05G004700	Gbar_D08G025320
137	GhirD05G006320	Gbar_D08G027420
138	GhirD05G006510	Gbar_D09G025260
139	GhirD05G009520	Gbar_D10G017760
140	GhirD05G010720	Gbar_D10G018680
141	GhirD05G013890	Gbar_D10G024800
142	GhirD05G020630	Gbar_D11G018640
143	GhirD05G022980	Gbar_D11G037370
144	GhirD05G035680	Gbar_D12G003660
145	GhirD05G036140	Gbar_D12G025880
146	GhirD06G001490	Gbar_D12G026140
147	GhirD06G003670	Gbar_D12G027390
148	GhirD06G006960	Gbar_D12G029520
149	GhirD06G007190	Gbar_D13G001910
150	GhirD06G009790	Gbar_D13G009410
151	GhirD06G011330	Gbar_D13G012000
152	GhirD06G011510	Gbar_D13G025430
153	GhirD06G019040	
154	GhirD06G020810	
155	GhirD07G000740	
156	GhirD07G001380	

157	GhirD07G003750
158	GhirD08G008860
159	GhirD08G010420
160	GhirD08G021830
161	GhirD08G022400
162	GhirD08G022450
163	GhirD08G024720
164	GhirD08G026690
165	GhirD08G027620
166	GhirD09G000190
167	GhirD09G005930
168	GhirD09G005950
169	GhirD09G016360
170	GhirD09G017180
171	GhirD09G018710
172	GhirD09G023500
173	GhirD09G023790
174	GhirD09G024000
175	GhirD09G024510
176	GhirD10G007110
177	GhirD10G017320
178	GhirD10G018140
179	GhirD10G019160
180	GhirD10G023090
181	GhirD10G025150
182	GhirD10G026840
183	GhirD11G000640
184	GhirD11G004560
185	GhirD11G014270
186	GhirD11G018250
187	GhirD11G019740
188	GhirD11G021190
189	GhirD11G029420
190	GhirD11G029480
191	GhirD11G036460
192	GhirD12G003650
193	GhirD12G022280
194	GhirD12G026180
195	GhirD12G026410
196	GhirD12G026430
197	GhirD12G027660
198	GhirD13G002120

199	GhirD13G004500
200	GhirD13G009380
201	GhirD13G009570
202	GhirD13G012160
203	GhirD13G021720
204	GhirD13G025380

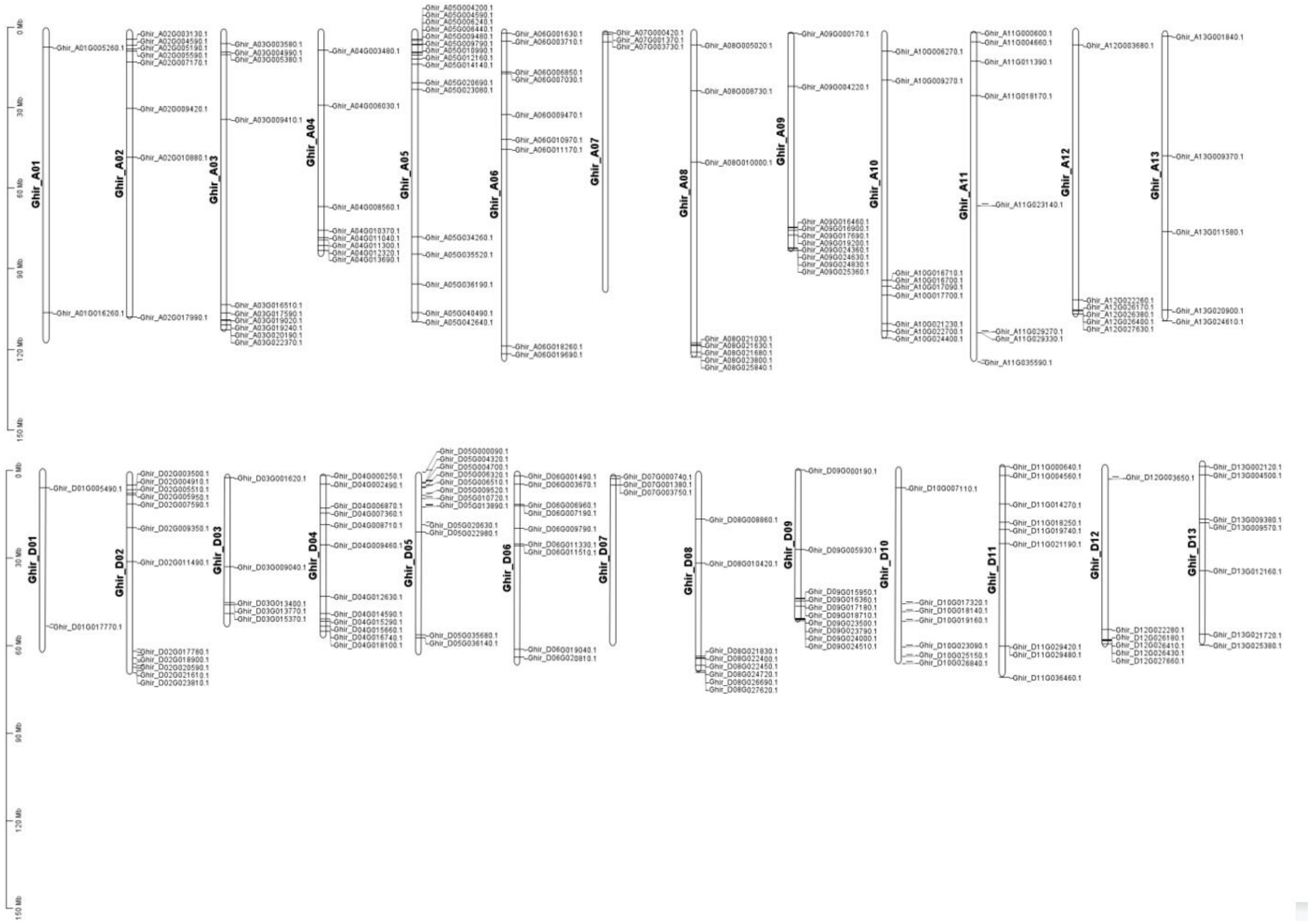

Figure S1. Chromosomal distribution of PP2C genes in *G. hirsutum*.

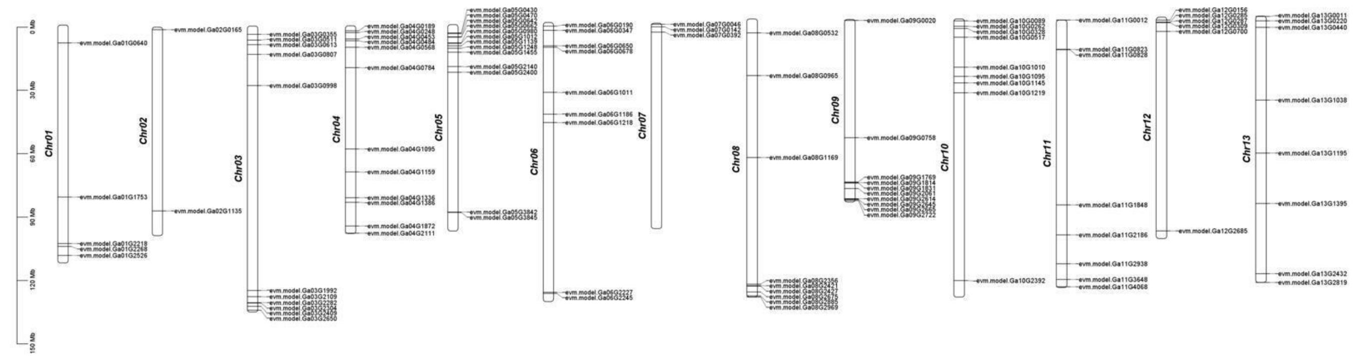

Figure S2. Chromosomal distribution of PP2C genes in *G. arboreum*.

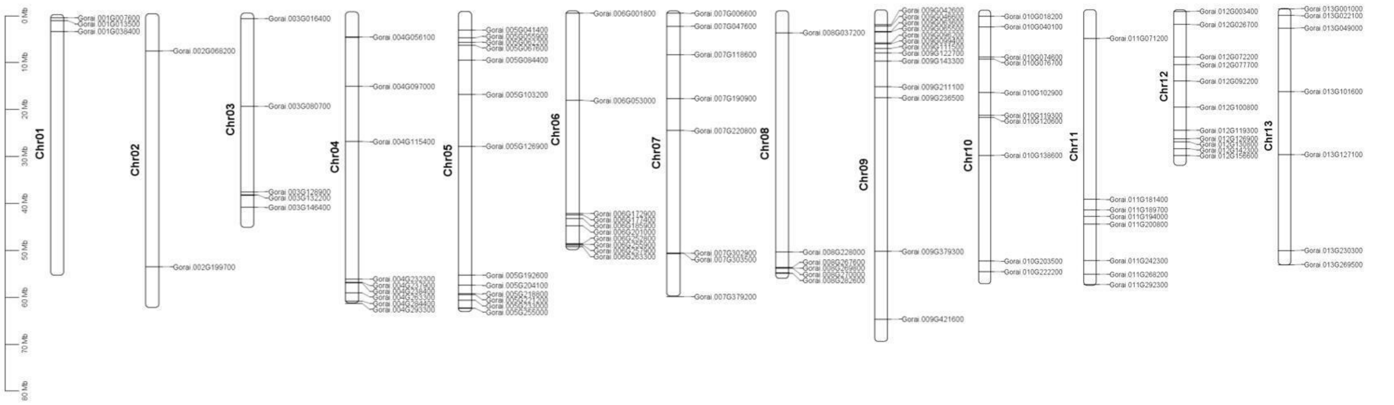

Figure S3. Chromosomal distribution of PP2C genes in *G. raimondii*.

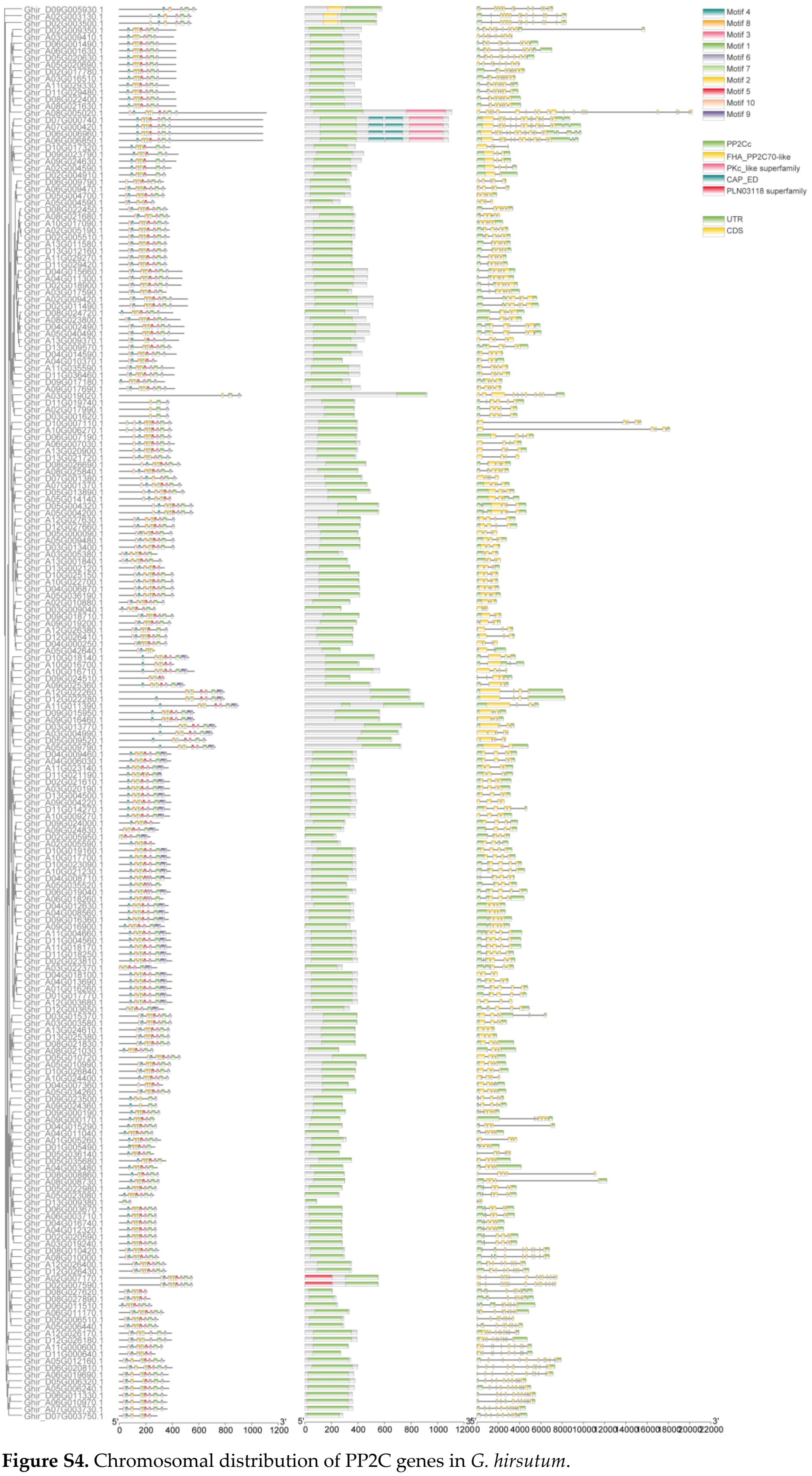

Figure S4. Chromosomal distribution of PP2C genes in *G. hirsutum*.

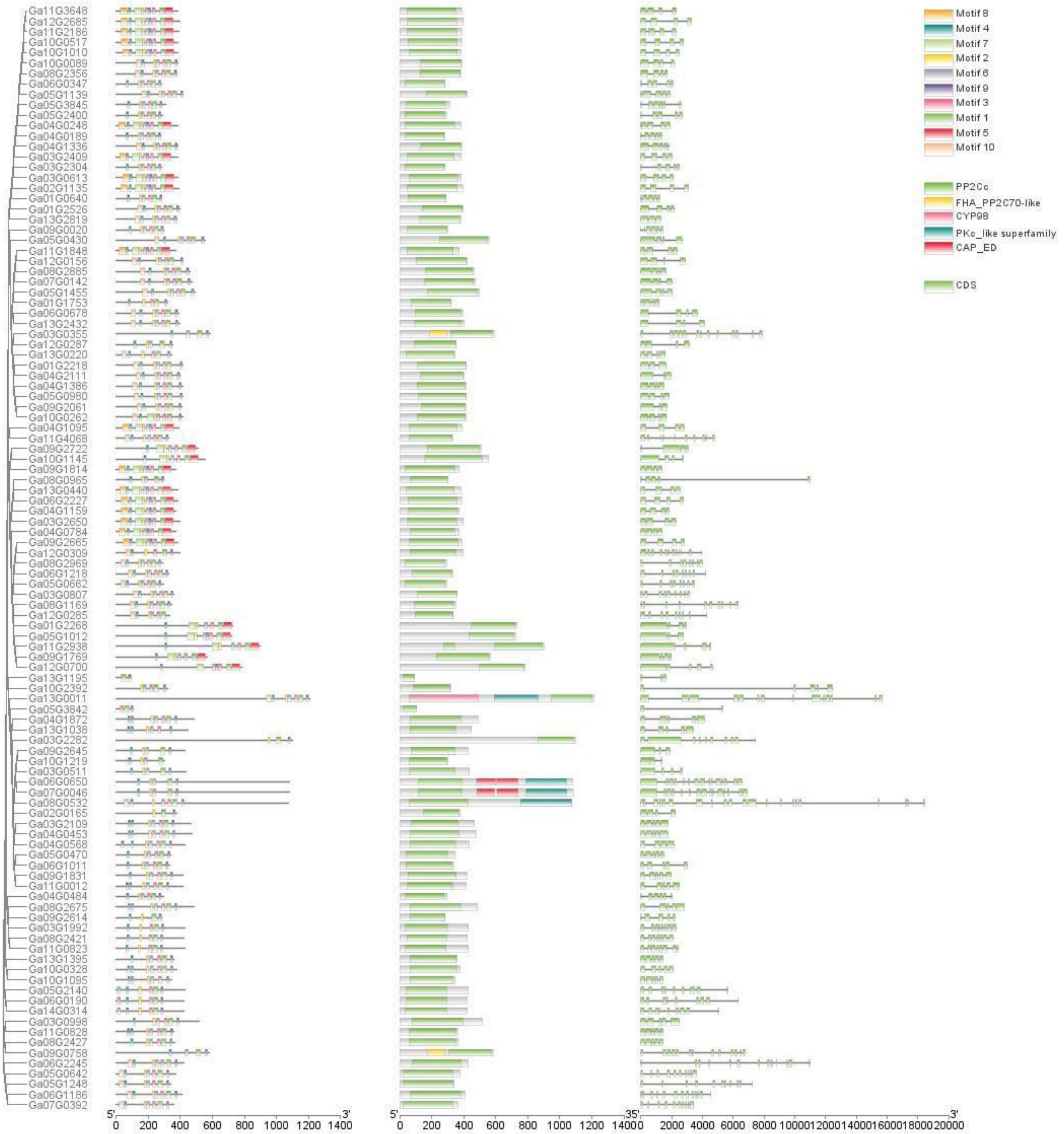

Figure S5. Chromosomal distribution of PP2C genes in *G. arboreum*.

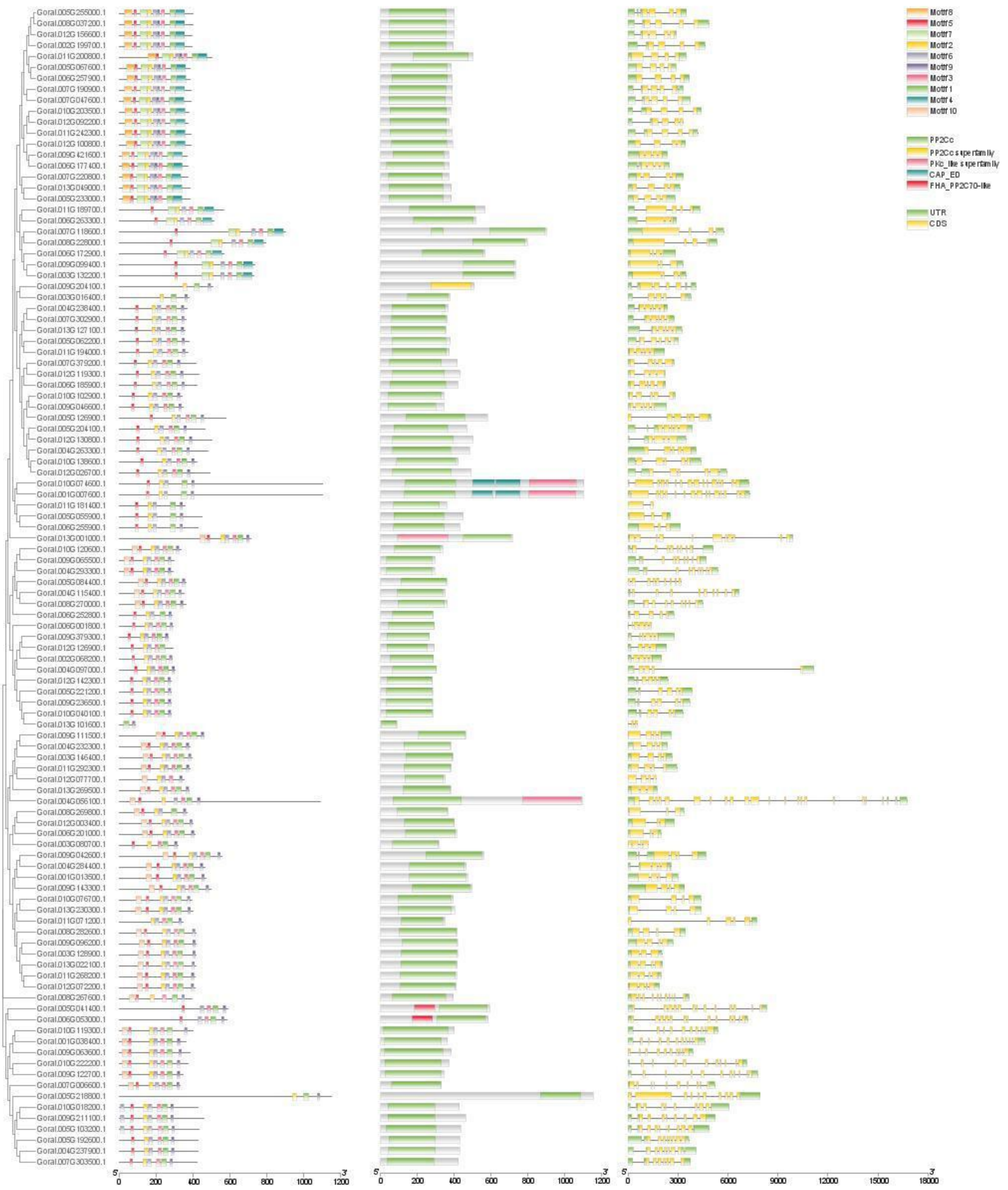

Figure S6. Chromosomal distribution of PP2C genes in *G. raimondii*.

Table S3. Characteristics of PP2C genes in *G. barbadense*.

Gene ID	DNA			Protein			Subcellular location	
	Position	CDS	GC content (%)	Size (aa)	Molecular weight (Da)	Isoelectric point	WolF PSPORT[a]	TargetP[b]
Gbar_A01G005250	A01:7451976-7455632	861	43.4	286	31129.21	4.98	cyto: 10, nucl: 2, mito: 1, E.R.: 1	T
Gbar_A01G016670	A01:104064855-104069239	1179	46.5	392	43570.65	8.94	chlo: 11, mito: 2, cyto: 1	N
Gbar_A02G002950	A02:3509129-3517619	1632	42.8	543	59618.51	5.72	cyto: 6.5, cyto_nucl: 6.5, nucl: 5.5, chlo: 1, vacu: 1	N
Gbar_A02G004480	A02:5975947-5979628	1179	42.8	392	42796.51	4.87	cyto: 7, nucl: 4, chlo: 2, vacu: 1	N
Gbar_A02G005470	A02:7950279-7953329	1152	46.3	383	42183	7.02	chlo: 4, mito: 4, nucl: 2, cyto: 2, E.R.: 1, pero: 1	N
Gbar_A02G006990	A02:11951852-11959381	1662	45.7	553	60441	5.14	chlo: 8, nucl: 3, cyto: 2, golg: 1	N
Gbar_A02G009130	A02:27664564-27670444	1548	44.1	515	56767	5.44	nucl: 10, chlo: 2, cyto: 1, cysk: 1	N
Gbar_A02G010690	A02:45157098-45158297	1023	48.5	340	36555	5.78	cyto: 12, chlo: 1, plas: 1	N
Gbar_A02G017570	A02:98997064-99001382	1143	46.2	380	40576	6.06	E.R.: 4.5, E.R._plas: 3.5, cyto: 3, golg: 2, plas: 1.5, chlo: 1, nucl: 1, mito: 1	N
Gbar_A03G003560	A03:4502901-4505648	1185	48.4	394	42200	6.95	cyto: 5, chlo: 3, mito: 2, cysk: 2, nucl: 1, plas: 1	C
Gbar_A03G004990	A03:7522755-7526153	2193	46.4	730	81326.16	5.41	nucl: 7, chlo: 3, mito: 2, plas: 2	N
Gbar_A03G005430	A03:8472545-8474175	1209	51.7	402	44253	7.83	nucl: 12, chlo: 1, cysk: 1	C
Gbar_A03G009420	A03:31661870-31674712	1284	43	427	46621	5.75	nucl: 6, chlo: 3, mito: 3, cyto: 1, plas: 1	N
Gbar_A03G016540	A03:95300041-95303596	1287	46.5	428	45837	8.75	cyto: 5, chlo: 3, cysk: 3, plas: 2, nucl: 1	N
Gbar_A03G017650	A03:98202044-98206081	1401	45.5	466	51450	5.33	mito: 5, chlo: 3, nucl: 3, cyto: 2, plas: 1	C
Gbar_A03G019300	A03:100840971-100844699	846	46.1	281	30842	8.77	chlo: 7, extr: 4, nucl: 2, cyto: 1	N
Gbar_A03G020270	A03:102311529-102314392	1152	44.5	383	42722	8.69	cyto: 10, nucl: 2, plas: 1, cysk: 1	N
Gbar_A03G022480	A03:104284451-104287925	1194	45.7	397	44027	8.36	chlo: 11, mito: 2, cyto: 1	N
Gbar_A04G003160	A04:7269098-7273103	864	42.5	287	31406	6.16	chlo: 14	T
Gbar_A04G005620	A04:26053192-26056612	1167	43.1	388	43639	6.84	nucl: 10, cyto: 2, plas: 1, cysk: 1	C
Gbar_A04G008170	A04:63156200-63158756	1110	43.2	369	41176	8.97	chlo: 8, nucl: 3, cyto: 2, cysk: 1	N

Gbar_A04G010130	A04:71845966-71848651	1293	47.6	430	47505	7.19	nucl: 7, chlo: 2, mito: 2, cyto: 1, plas: 1, pero: 1	N
Gbar_A04G010830	A04:74397806-74400288	846	44.4	281	30791	7.21	chlo: 13, nucl: 1	N
Gbar_A04G011130	A04:75245424-75249381	1425	46.1	474	52695	5.52	cyto: 9, nucl: 2, chlo: 1, mito: 1, E.R.: 1	N
Gbar_A04G012230	A04:77366496-77368954	843	47.2	280	30419	8.27	cyto: 8.5, cyto_nucl: 6.5, nucl: 3.5, chlo: 1, vacu: 1	N
Gbar_A04G013560	A04:78942518-78945433	1191	46.9	396	44371	8.73	nucl: 6, cyto: 5, chlo: 2, vacu: 1	N
Gbar_A05G003650	A05:3592924-3597068	1677	44.2	558	60868	4.67	cyto: 9, chlo: 1, nucl: 1, plas: 1, pero: 1, cysk: 1	N
Gbar_A05G004030	A05:3922897-3924746	1032	44.6	343	37454	5.7	chlo: 6, mito: 4, nucl: 1, cyto: 1, E.R.: 1, pero: 1	T
Gbar_A05G005780	A05:5405700-5410710	1125	43	374	40901	5.55	vacu: 5, golg: 4, chlo: 3, cyto: 1, mito: 1	N
Gbar_A05G005970	A05:5536470-5539948	885	44.7	294	31815	5.32	nucl: 9, cyto: 2, chlo: 1, mito: 1, cysk: 1	C
Gbar_A05G008950	A05:8131873-8134534	1251	50.4	416	454110	6.07	chlo: 10, cyto: 2, plas: 1, vacu: 1	C
Gbar_A05G009240	A05:8341272-8345680	2169	44	722	80802	5.72	chlo: 12, nucl: 1, mito: 1	N
Gbar_A05G010390	A05:9470673-9473508	849	45.5	282	30780	5.41	cyto: 8, nucl: 4, cysk: 2	N
Gbar_A05G011560	A05:10587958-10596306	1032	43.3	343	37736	5.18	nucl: 7.5, cyto_nucl: 5, chlo: 2, cysk: 2, cyto: 1.5, plas: 1	N
Gbar_A05G013540	A05:12527049-12530247	1485	44.8	494	53356	5.04	nucl: 14	N
Gbar_A05G020000	A05:19143107-19147049	1287	44.6	428	46244	5.56	chlo: 14	N
Gbar_A05G022350	A05:21707557-21711210	876	44.6	291	31925	9.18	chlo: 11.5, chlo_mito: 7.5, mito: 2.5	N
Gbar_A05G025310	A05:26211108-26212341	540	45.2	179	20260	9.01	chlo: 9, cyto: 3, nucl: 1, cysk: 1	C
Gbar_A05G033200	A05:72539986-72541690	1173	47.1	390	42085	6.67	chlo: 8, mito: 3, cyto: 1, E.R.: 1, golg: 1	C
Gbar_A05G034590	A05:78961952-78963778	1113	43.2	370	41698	9.27	mito: 5, cyto: 2, extr: 2, chlo: 1, nucl: 1, plas: 1, vacu: 1, golg: 1	N
Gbar_A05G035290	A05:88740558-88742514	1242	48.9	413	45391	5.52	cyto: 7, nucl: 6, chlo: 1	N
Gbar_A05G039550	A05:99003978-99009744	1464	43.2	487	53735	5.33	nucl: 8, chlo: 3, mito: 1.5, cyto_mito: 1.5, cyto: 1	C
Gbar_A05G041700	A05:102322359-102324503	792	46	263	29129	6.47	nucl: 5, cyto: 4, chlo: 3, mito: 1, cysk: 1	N
Gbar_A06G001530	A06:1531381-1538965	1281	44.3	426	46213	5.54	mito: 7.5, cyto_mito: 4.5, chlo: 4, nucl: 2	N
Gbar_A06G003570	A06:4446551-4449947	846	41.8	281	30873	7.74	chlo: 3, plas: 3, E.R.: 3, nucl: 2, cyto: 1, extr: 1, vacu: 1	N
Gbar_A06G006290	A06:11849980-11851743	543	44.4	180	20323	9.36	chlo: 4, nucl: 3, cysk: 3, mito: 2, cyto: 1, vacu: 1	N
Gbar_A06G006780	A06:14906546-14916113	3249	44	1082	119734	5.25	cyto: 7, nucl: 4.5, nucl_plas: 3.5, plas: 1.5, cysk: 1	N

Gbar_A06G006990	A06:15495535-15500170	1182	50.3	393	43030	5.31	nucl: 7.5, cyto_nucl: 5.5, chlo: 3, cyto: 2.5, plas: 1	N
Gbar_A06G009380	A06:29788021-29791082	1023	46.8	340	37285	6.6	nucl: 12, chlo: 1, cysk: 1	N
Gbar_A06G010870	A06:38542094-38547518	1083	43.9	360	39782	4.98	nucl: 10.5, cyto_nucl: 6, chlo: 1, cysk: 1, golg: 1	N
Gbar_A06G011060	A06:42133137-42137494	1011	43.7	336	36547	5.17	chlo: 9, nucl: 4, cyto: 1	C
Gbar_A06G015900	A06:103884790-103887364	1092	45.7	363	40828	8.92	chlo: 3, plas: 3, E.R.: 3, nucl: 2, cyto: 2, extr: 1	C
Gbar_A06G018290	A06:109798780-109801542	1161	45	386	42860	8.64	cyto: 4, chlo: 2, nucl: 2, extr: 2, golg: 2, vacu: 1, E.R.: 1	N
Gbar_A06G019700	A06:112322876-112330144	1113	44.6	370	40499	4.77	nucl: 6, chlo: 3, cyto: 3, mito: 2	N
Gbar_A07G000770	A07:790686-800445	3252	45	1083	119712	5.06	nucl: 7.5, cyto_nucl: 5.5, cyto: 2.5, chlo: 2, mito: 1, golg_plas: 1	N
Gbar_A07G001400	A07:1583796-1588240	1416	43.4	471	51959	5.58	chlo: 7, nucl: 3, cyto: 1, mito: 1, extr: 1, vacu: 1	N
Gbar_A07G003520	A07:4144266-4148809	1086	43.1	361	39751	5.01	chlo: 10, mito: 3, nucl: 1	N
Gbar_A08G005010	A08:6093350-6114918	3276	41.8	1091	122487	5.83	cyto: 6, cysk: 3, chlo: 1, nucl: 1, plas: 1, extr: 1, golg: 1	E
Gbar_A08G008830	A08:22479918-22491802	906	45.1	301	32711	8.91	chlo: 14	N
Gbar_A08G010130	A08:48266903-48273554	897	42.5	298	32770	5.05	nucl: 13, cyto: 1	T
Gbar_A08G021640	A08:113805440-113808343	1146	46.9	381	40669	8.17	nucl: 5.5, cyto_nucl: 4, chlo: 3, cysk: 3, cyto: 1.5, plas: 1	C
Gbar_A08G022250	A08:114593037-114597126	1287	47.5	428	46164	8.46	chlo: 5, mito: 5, nucl: 2, extr: 1, pero: 1	N
Gbar_A08G022310	A08:114689726-114692704	1095	45	364	40566	7.98	chlo: 9, cyto: 3, plas: 1, vacu: 1	N
Gbar_A08G024670	A08:117260770-117264804	1452	45.9	483	53551	5.33	chlo: 10, nucl: 2, mito: 1, plas: 1	C
Gbar_A08G026740	A08:119170634-119174275	1098	43.5	365	40410	8.02	nucl: 8, mito: 3, chlo: 1, cyto: 1, cysk: 1	N
Gbar_A08G027500	A08:119761338-119766782	705	43.8	234	25380	6.07	cyto: 8, chlo: 3, mito: 2, plas: 1	N
Gbar_A09G000220	A09:496160-498213	840	41.7	279	31384	5.57	nucl: 5, chlo: 4, cyto: 1, mito: 1, plas: 1, extr: 1, cysk: 1	N
Gbar_A09G006430	A09:45759601-45766751	1755	43.5	584	64804	5.5	cyto: 6, cysk: 4, chlo: 1, nucl: 1, extr: 1, golg: 1	E
Gbar_A09G016730	A09:68782212-68784202	1701	41.9	566	62788	5.24	chlo: 14	C
Gbar_A09G017180	A09:69108104-69109462	1116	44.2	371	41047	8.91	chlo: 7, nucl: 4, mito: 2, pero: 1	N
Gbar_A09G017940	A09:69928572-69930829	1257	43.8	418	46269	5.78	chlo: 11.5, chlo_mito: 7, mito: 1.5, cysk: 1	N
Gbar_A09G019420	A09:71645123-71647237	1236	46.3	411	44812	6.49	nucl: 6, cyto: 5, mito: 1, plas: 1, cysk: 1	N

Gbar_A09G024850	A09:76403331-76406411	1284	41.6	427	47086	4.91	nucl: 6.5, cyto_nucl: 5, chlo: 4, cyto: 2.5, cysk: 1	N
Gbar_A09G025060	A09:76533767-76537531	1158	46.7	385	42363	6.81	chlo: 4, nucl: 3.5, cysk_nucl: 2.5, plas: 2, cyto: 1, mito: 1, vacu: 1, E.R.: 1	N
Gbar_A09G025580	A09:77047327-77050576	1482	42.6	493	53321	4.9	chlo: 4, cyto: 3, nucl: 2, extr: 2, vacu: 2, mito_plas: 1	N
Gbar_A10G006990	A10:7720622-7738479	1098	48.3	365	39335	6.2	nucl: 6, cyto: 4, chlo: 2, plas: 1, cysk: 1	C
Gbar_A10G011450	A10:23585756-23587759	921	37.6	306	33613	4.53	nucl: 7, chlo: 3, mito: 2, cyto: 1, plas: 1	N
Gbar_A10G017640	A10:87458020-87462701	1647	41.8	548	60040	5.71	cyto: 6, cysk: 3, chlo: 2, plas: 2, nucl: 1	N
Gbar_A10G018030	A10:89754245-89756584	1119	43.2	372	41558	6.52	chlo: 11, mito: 2, cyto: 1	N
Gbar_A10G018520	A10:92871657-92875376	1152	46.6	383	42363	8.77	chlo: 13, mito: 1	N
Gbar_A10G022390	A10:104125293-104133001	1161	43.7	386	42840	5.97	chlo: 13, extr: 1	N
Gbar_A10G023900	A10:107173447-107175432	1230	47.6	409	44647	5.1	nucl: 11, cyto: 1, plas: 1, cysk: 1	N
Gbar_A10G025590	A10:109884575-109887565	1161	44.3	386	42339	7.94	cyto: 6, chlo: 4, nucl: 1, plas: 1, cysk: 1, E.R._vacu: 1	C
Gbar_A11G000180	A11:179900-185076	987	46.6	328	36023	8.2	chlo: 7, nucl: 3, cyto: 3, cysk: 1	N
Gbar_A11G004230	A11:3555226-3559564	1164	46.5	387	43234	9.08	chlo: 8, mito: 3, nucl: 2, cyto: 1	N
Gbar_A11G011090	A11:10635903-10641632	2340	43.2	779	86772.43	5.96	chlo: 14	C
Gbar_A11G017710	A11:22627026-22630878	1167	45.9	388	43452	8.96	cyto: 5, cysk: 4, mito: 3, nucl: 1, E.R.: 1	M
Gbar_A11G022510	A11:59674424-59677839	1113	45.8	370	41045	5.88	mito: 5, cyto: 2, extr: 2, chlo: 1, nucl: 1, plas: 1, vacu: 1, golg: 1	N
Gbar_A11G028830	A11:101482499-101485271	1083	45.5	360	40103	6.64	cyto: 8, nucl: 5, chlo: 1	N
Gbar_A11G028880	A11:101590098-101593971	1287	45.5	428	45970	5.98	nucl: 10, chlo: 2, mito: 1, golg_plas: 1	N
Gbar_A11G035000	A11:113052529-113055861	1248	44.1	415	46025	5.49	cysk: 8, mito: 3, cyto: 2, nucl: 1	N
Gbar_A12G003590	A12:6176612-6179903	1194	45	397	43844	8.39	nucl: 8, mito: 4, chlo_mito: 2.83333, cyto_mito: 2.83333, cyto: 1	N
Gbar_A12G026190	A12:99697774-99703434	1029	43.1	342	37698.71	6.21	chlo: 5, nucl: 5, cyto: 2, mito: 1, cysk: 1	C
Gbar_A12G027500	A12:100785500-100789082	1260	52.4	419	45890.76	6.39	mito: 7.5, cyto_mito: 4.5, chlo: 3, nucl: 3	M
Gbar_A13G009830	A13:47379409-47382813	1347	43.1	448	49685.13	5.87	chlo: 3, plas: 3, E.R.: 3, nucl: 2, cyto: 1, extr: 1, vacu: 1	C
Gbar_A13G012360	A13:76709031-76711588	1080	45.6	359	39999.34	6.42	cyto: 9, chlo: 1, nucl: 1, extr: 1, cysk: 1, E.R._vacu: 1	C
Gbar_A13G024830	A13:109031311-109032873	1143	45.1	380	41261.1	8.17	cysk: 4, chlo: 3, nucl: 3, mito: 2, cyto: 1, pero: 1	C
Gbar_D01G005440	D01:6892640-6893874	861	43.6	286	31195.22	5.17	nucl: 6, cyto: 5, nucl_plas: 5, plas: 2, cysk: 1	N

Gbar_D02G003600	D02:4684789-4693197	1632	43.3	543	59734.57	5.72	nucl: 9, chlo: 3, cyto: 1, plas: 1	N
Gbar_D02G005020	D02:6506142-6509902	1263	42.5	391	42722.44	4.73	nucl: 11, chlo: 1, cyto: 1, cysk: 1	N
Gbar_D02G005650	D02:7451247-7454007	1134	43.6	377	41413.222	7.57	chlo: 6, cyto: 5, cysk: 2, mito: 1	C
Gbar_D02G006190	D02:18939291-18955522	1038	43.1	383	42325.23	7.33	chlo: 3, plas: 3, E.R.: 3, nucl: 2, cyto: 2, extr: 1	C
Gbar_D02G009810	D02:30226676-30232408	1548	44.5	427	46615.35	5.33	cyto: 4, chlo: 2, nucl: 2, extr: 2, golg: 2, vacu: 1, E.R.: 1	C
Gbar_D02G012000	D02:59518767-59522582	1287	47.2	515	56627.24	5.5	nucl: 9, chlo: 2, cyto: 2, mito: 1	N
Gbar_D02G018420	D02:61567999-61569764	1401	46.1	428	45720.19	8.44	cyto: 8, nucl: 2, extr: 2, chlo: 1, cysk: 1	C
Gbar_D02G019500	D03:1161154-1164889	1128	46.6	466	51421.91	5.49	chlo: 14	C
Gbar_D03G001670	D03:43013453-43015400	1251	51.6	375	40010	6	cyto: 8, mito: 3, E.R.: 1, golg: 1	P
Gbar_D03G012870	D03:43810963-43814361	2193	46.7	416	45813	8.09	nucl: 6.5, cyto_nucl: 6, cyto: 4.5, chlo: 1, plas: 1, E.R._vacu: 1	N
Gbar_D03G013270	D03:46618562-46622666	903	47.7	730	81300.21	5.41	chlo: 9.5, chlo_mito: 6.5, mito: 2.5, nucl: 2	C
Gbar_D03G014860	D04:3201420-3207225	1470	43.3	394	42151.78	6.9	nucl: 6, chlo: 3, mito: 3, cyto: 1, golg_plas: 1	N
Gbar_D04G002400	D04:11055706-11057637	1242	49	489	53950.58	5.47	chlo: 2, cyto: 2, extr: 2, golg: 2, mito: 1, E.R.: 1	C
Gbar_D04G006830	D04:16758830-16762412	1113	43.6	413	45479.8	5.61	nucl: 7.5, cyto_nucl: 5.5, cyto: 2.5, chlo: 2, mito: 1, golg_plas: 1	N
Gbar_D04G008700	D04:23260293-23263820	1170	43.1	370	41684.07	9.27	cyto: 4, plas: 2.5, E.R._plas: 2.5, vacu: 2, E.R.: 1.5, mito: 1, extr: 1, pero: 1, golg: 1	C
Gbar_D04G009560	D04:38239380-38241947	912	43.3	388	43701.81	6.84	plas: 6, nucl: 3, chlo: 2, E.R.: 2, cyto: 1	P
Gbar_D04G012670	D04:46584722-46590392	1425	46.3	369	41149.27	9.1	cyto: 6, chlo: 3, nucl: 2, cysk: 2, plas: 1	C
Gbar_D04G015860	D04:48136432-48138892	840	47	474	52548	5.32	cyto: 7, cysk: 3, nucl: 2, chlo: 1, E.R._vacu: 1	C
Gbar_D04G016910	D04:49750463-49753377	1191	46.9	279	30338.29	8.27	chlo: 7, cyto: 3, nucl: 2, mito: 2	C
Gbar_D04G018240	D05:3381366-3385967	1677	44.3	396	44323.51	8.71	cyto: 3.5, chlo: 3, cyto_nucl: 3, mito: 2, extr: 2, nucl: 1.5, plas: 1, vacu: 1	C
Gbar_D05G004150	D05:4971869-4977050	1125	43.5	558	60738.22	4.69	pero: 8, cyto: 3, nucl: 2, golg: 1	P
Gbar_D05G006210	D05:5094866-5098315	885	45.5	374	40854.9	5.11	chlo: 11, mito: 2, nucl: 1	C
Gbar_D05G006400	D05:7620840-7623689	1254	50.9	294	31767.2	5.32	nucl: 10, chlo: 2, cyto: 1, vacu: 1	N
Gbar_D05G009430	D05:7818225-7821395	2199	44.4	417	45464.6	5.83	nucl: 6, cyto: 5, cysk: 2, chlo: 1	C
Gbar_D05G009690	D05:8924780-8928603	1386	46	732	81885.03	5.57	cyto: 6, cysk: 3, plas: 2, chlo: 1, nucl: 1, golg: 1	C

Gbar_D05G010980	D05:10001132-10008798	1032	43.3	461	49942.06	8.9	nucl: 5, chlo: 4, mito: 4, cyto: 1	N
Gbar_D05G011960	D05:11860166-11864129	1485	44.9	343	37607.25	5.17	chlo: 3, vacu: 3, cyto: 2, mito: 2, plas: 2, nucl: 1, E.R.: 1	C
Gbar_D05G013980	D05:17833052-17837041	1287	44.4	494	53452.96	5	chlo: 9, mito: 2, nucl: 1, cyto: 1, pero: 1	C
Gbar_D05G020660	D05:20304188-20307708	846	44.9	428	46244.59	5.56	cysk: 6, cyto: 4, chlo: 1, nucl: 1, extr: 1, golg: 1	C
Gbar_D05G023010	D06:4066328-4070049	846	41.7	281	30703.62	8.87	chlo: 14	C
Gbar_D06G003750	D06:10861567-10867761	3249	44	281	30981.01	7.09	chlo: 6, plas: 3, nucl: 2, cyto: 1, extr: 1, pero: 1	C
Gbar_D06G007000	D06:57352392-57367438	1176	45.5	1082	119788.85	5.14	chlo: 6, nucl: 6, mito: 1, pero: 1	C
Gbar_D06G018850	D07:1434484-1437920	1416	43.5	391	43358.28	8.16	chlo: 12, mito: 1, cysk: 1	C
Gbar_D07G001400	D08:30377000-30383686	900	42.6	471	51939.67	5.82	nucl: 5, cyto: 5, chlo: 1, mito: 1, pero: 1, cysk: 1	N
Gbar_D08G011020	D08:60349492-60352237	1146	47.6	299	32901.78	5.01	nucl: 5, chlo: 4, cyto: 4, cysk: 1	N
Gbar_D08G022400	D08:61161051-61164042	1095	44.7	381	40684.12	7.88	cyto: 10, chlo: 3, nucl: 1	C
Gbar_D08G023030	D08:63502648-63506755	1215	45.8	364	40659.15	8.01	chlo: 9, nucl: 2, E.R.: 2, plas: 1	C
Gbar_D08G025320	D08:65322516-65325543	1392	44.3	483	53588.28	5.39	chlo: 4, nucl: 4, cyto: 2, mito: 2, pero: 2	C
Gbar_D08G027420	D09:50648956-50652112	1482	42.8	463	51220.8	6.34	cyto: 3, vacu: 3, E.R.: 3, nucl: 2, golg: 2, chlo: 1	C
Gbar_D09G025260	D10:47279158-47283609	1578	41	493	53366.89	4.95	nucl: 5, chlo: 2, cyto: 2, mito: 2, plas: 1.5, golg_plas: 1.5, cysk: 1	N
Gbar_D10G017760	D10:50717273-50720758	1152	46.5	525	57422.93	5.47	cyto: 6.5, cyto_nucl: 5.5, nucl: 3.5, chlo: 1, plas: 1, extr: 1, cysk: 1	C
Gbar_D10G018680	D10:62543069-62545026	1236	48.1	383	42228.02	8.77	cyto: 5, nucl: 4, chlo: 3, cysk: 2	C
Gbar_D10G024800	D11:19490635-19494398	1155	45.5	411	45032.91	5.05	chlo: 4, cyto: 3, extr: 2, vacu: 2, nucl: 1, E.R.: 1, mito_plas: 1	C
Gbar_D11G018640	D11:26503019-26506497	1113	46	388	43432.44	8.96	nucl: 7.5, cyto_nucl: 5.5, chlo: 4, cyto: 2.5	N
Gbar_D11G037370	Scaffold919:9264-16036	1134	44.8	377	40238.18	6.15	chlo: 4, E.R.: 3, nucl: 2, cyto: 1, mito: 1, plas: 1, extr: 1, vacu: 1	C
Gbar_D12G003660	D12:4774171-4778883	1194	45.1	397	44034.09	8.4	nucl: 8, cysk: 4, cyto: 1, extr: 1	N
Gbar_D12G025880	D12:56474136-56478621	1185	44.1	394	43055.65	5.24	nucl: 11, chlo: 2, cyto: 1	N
Gbar_D12G026140	D12:56746696-56751408	840	43	279	30687.81	9.01	cyto: 8, plas: 2.5, nucl: 2, golg_plas: 2, chlo: 1	C
Gbar_D12G027390	D12:57774334-57778094	1254	51.9	417	45605.35	6.3	chlo: 6, cyto: 2, extr: 2, nucl: 1, vacu: 1, E.R.: 1, mito_plas: 1	C

Gbar_D12G029520	D12:25857-33840	1890	49.8	629	69172.86	4.94	mito: 5, cyto: 3, chlo: 1, nucl: 1, plas: 1, extr: 1, vacu: 1, golg: 1	M
Gbar_D13G001910	D13:1615249-1617351	1251	45.4	416	46126.73	6.76	chlo: 5, nucl: 4, cyto: 4, plas: 1	C
Gbar_D13G009410	D13:20490575-20495234	1185	43.2	394	43432.35	6.72	chlo: 13.5, chlo_mito: 7.5	C
Gbar_D13G012000	D13:34345822-34349203	1110	45.2	369	41386.04	7.18	chlo: 7, nucl: 5, cyto: 1, vacu: 1	C
Gbar_D13G025430	D13:60117146-60118786	1143	45.9	380	41202.07	7.9	cyto: 4, cysk: 4, chlo: 3, nucl: 3	C

[a] WoLF PSORT prediction abbreviations: chlo, chloroplast; cyto, cytoplasm; nucl, nucleus; plas, plastid membrane; cyto, cell solute; mito, mitochondria; extr, cell wall; cysk, cytoskeleton; E.R., endoplasmic reticulum; golg, golg body.

[b] Targt prediction abbreviations: N, nucleus; C, chloroplast; M, mitochondria; T, cytosol; E, endoplasmic reticulum; P, plastid membrane.

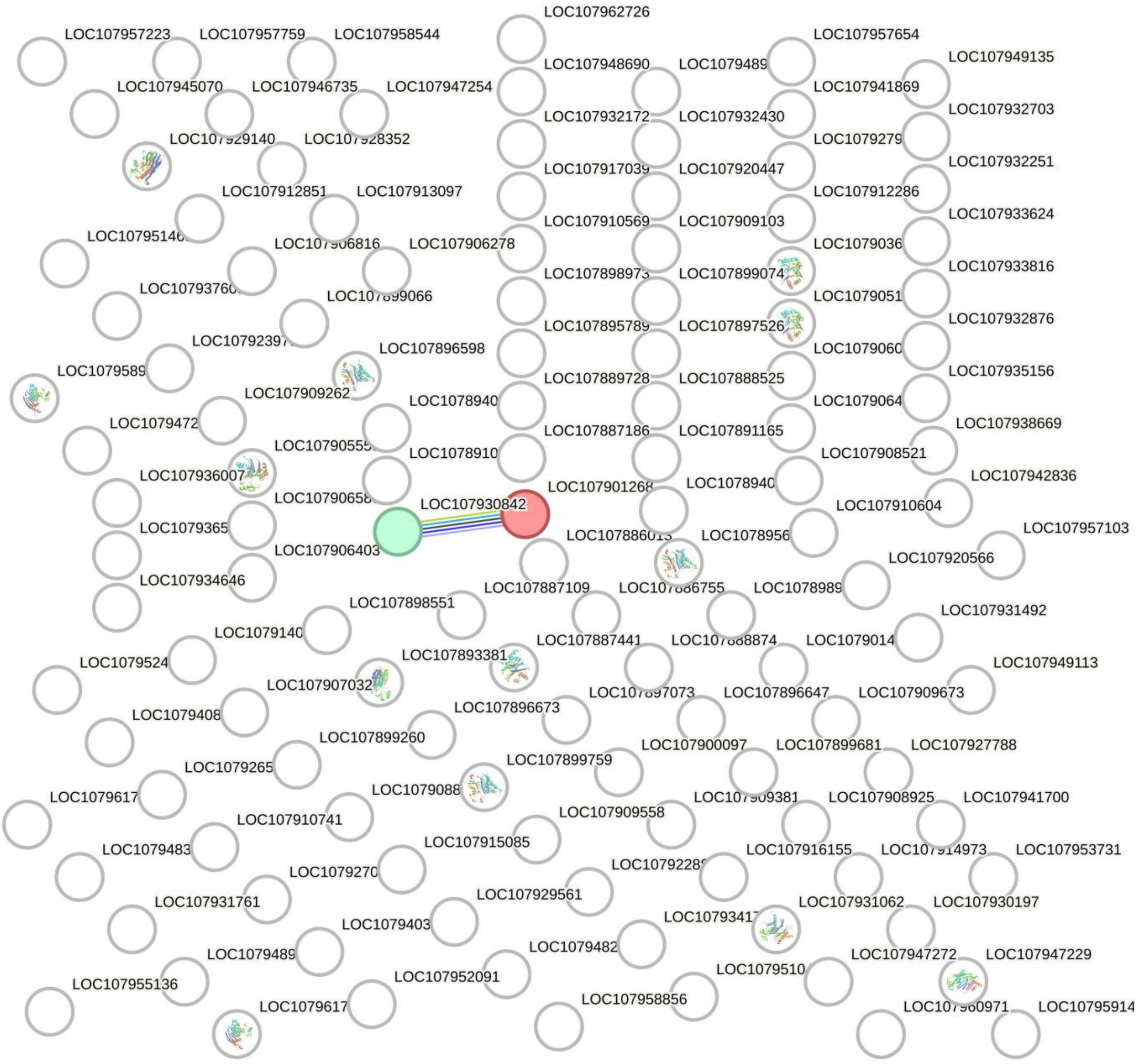

Figure S7. Predicted protein-protein interactive networks of GbPP2Cs based on *G. hirsutum* homologs. Nodes represent proteins (splice isoforms or post-translational modifications collapsed). Filled nodes represent proteins of known 3D structure. Edges represent protein-protein associations, i.e. proteins jointly contribute to a shared function. The blue-green and purple lines represent the known interactions obtained from curated databases and experimentally determined data, respectively. The dark green, red and dark blue lines represent the predicted interactions from gene neighborhood, gene fusions, and gene co-occurrence. The yellow-green, black and light blue lines represent the protein of textming, co-expression, and homology. The single interacting pair LOC107901268 (red, Gbar_A06G006780) and LOC107930842 (green, Gbar_A07G000770) is shown.

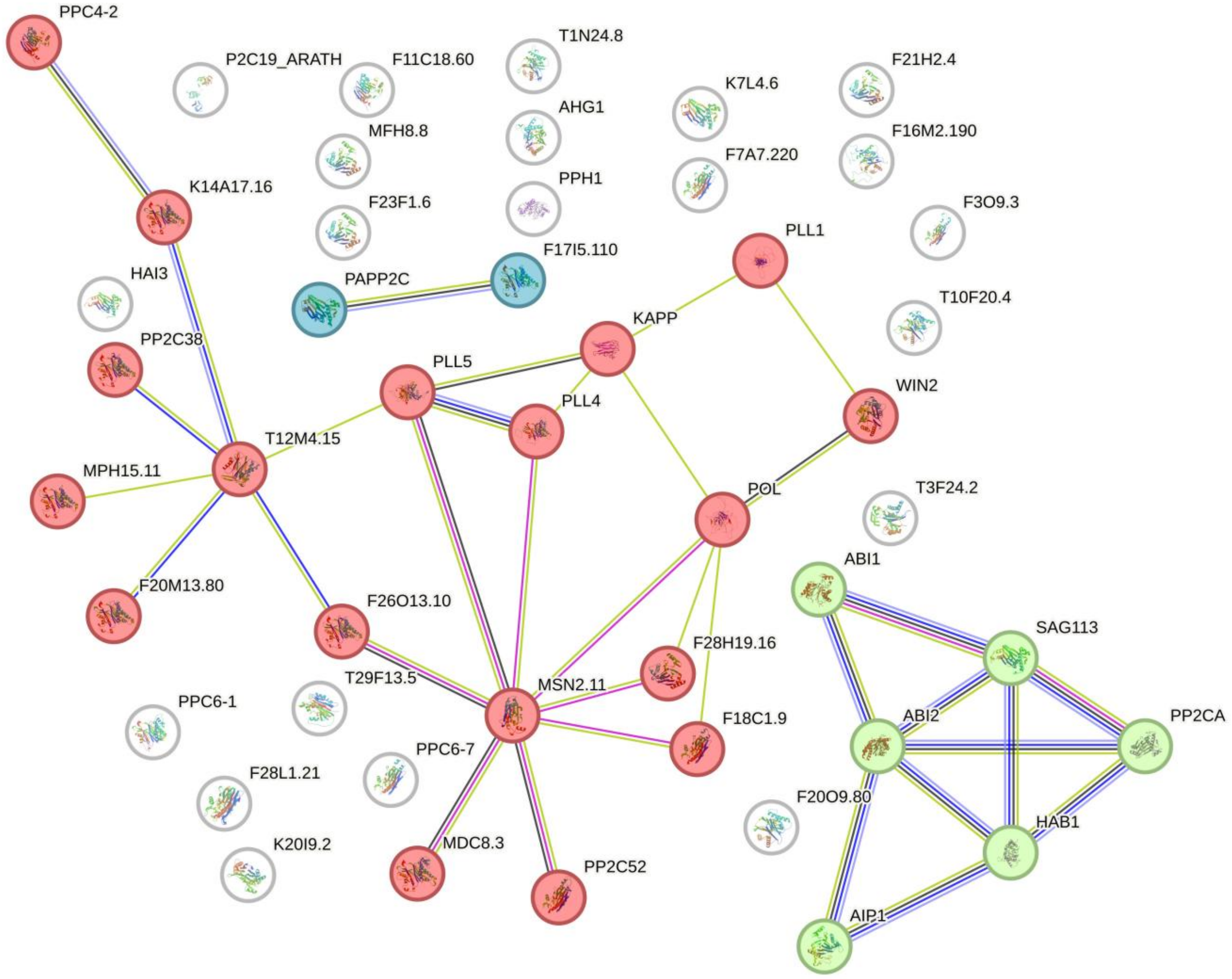

Figure S8. Predicted protein-protein interactive networks of GbPP2Cs based on *A. thaliana* homologs. Node and edge representations follow Figure S7. Three functional networks are shown: protein dephosphorylation (red, 18 PP2Cs), peptidyl-threonine dephosphorylation (green, 6 PP2Cs), and “F17I5.110 and PAPP2C” (blue, 2 PP2Cs).

Table S4. KEGG enrichment of GbPP2Cs.

Term ID	Description	Gene count	signal	FDR	GbPP2C proteins
ghi04016	MAPK signaling pathway-plant	15	2.55	8.25e-13	Gbar_A05G013540,Gbar_D03G012870,Gbar_D05G004150,Gbar_A10G006990,Gbar_A09G019420,Gbar_A12G027500,Gbar_D07G001400,Gbar_D08G027420,Gbar_A05G041700,Gbar_D12G027390,Gbar_A02G010690,Gbar_A05G035290,Gbar_A05G003650,Gbar_A05G008950,Gbar_A06G006990
ghi04075	Plant hormone signal transduction	15	1.66	2.73e-09	

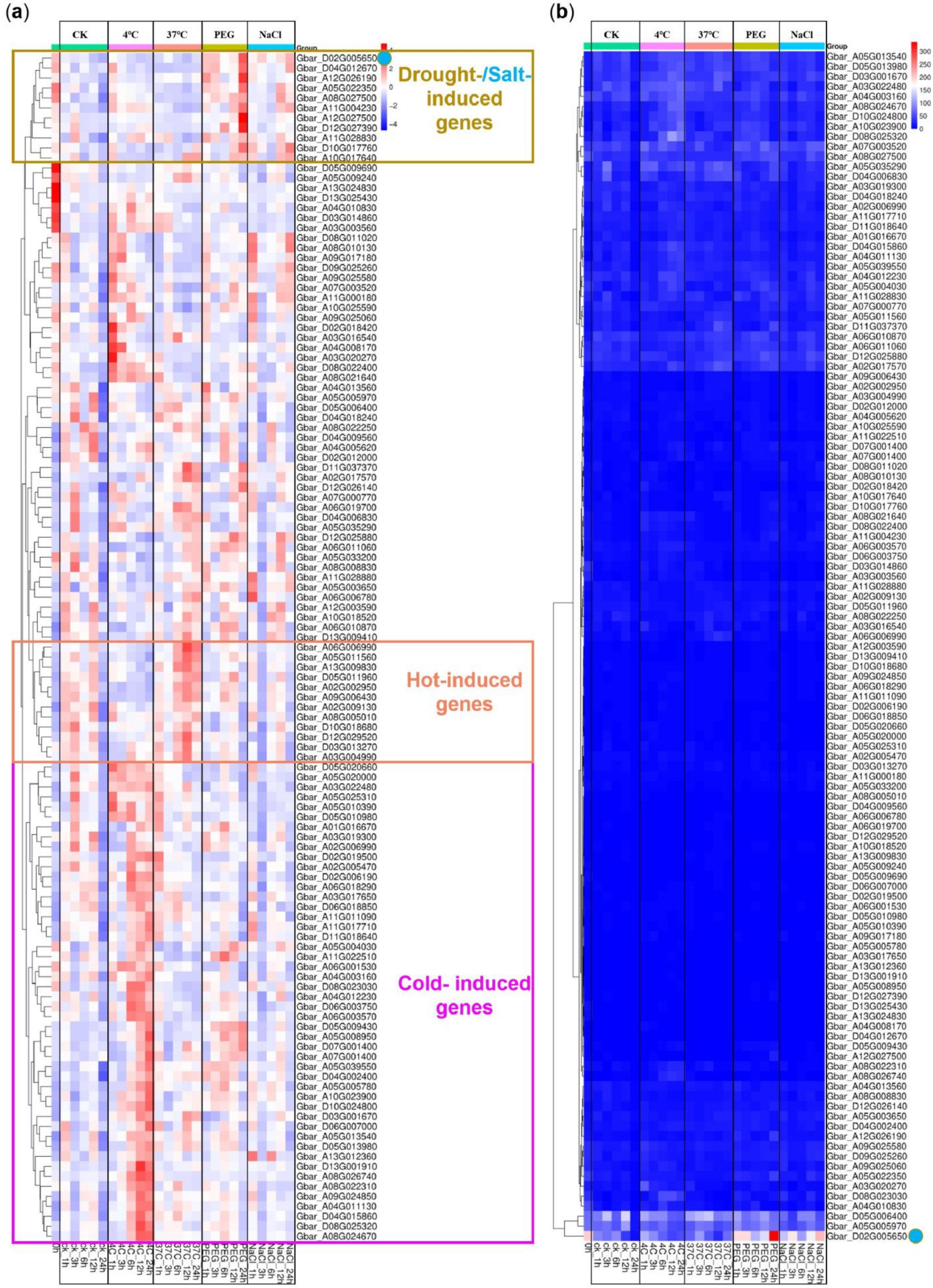

(a)
(b)
CK
4°C
37°C
PEG
NaCl
Group
Drought-/Salt-induced genes
Hot-induced genes
Cold- induced genes

Figure S9. Expression level of *GbPP2C* genes after cold (4℃), heat (37℃), drought (PEG), and salt (NaCl) treatments in *G. barbadense* accession H7124. (**a**) Z-scored FPKM values. (**b**) Raw FPKM values. Blue dot highlighted the optimal candidate, *Gbar_D02G005650*, induced by drought (PEG) and salt (NaCl) stress.

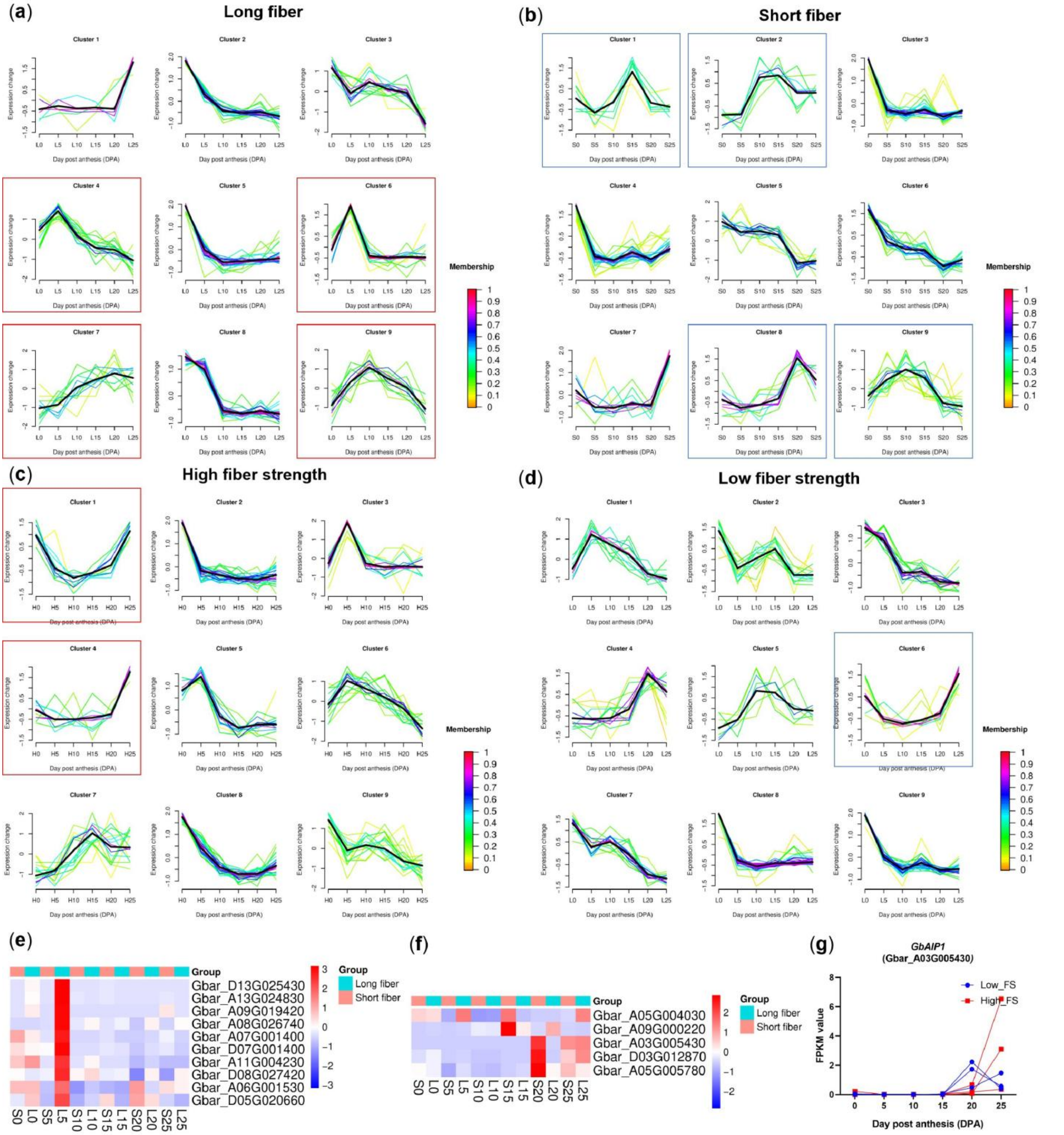

Figure S10. Identification of *GbPP2C* genes related to fiber quality. (**a**,**b**) Mfuzz time series of *GbPP2C* genes during different fiber development stages of long- and short-fiber G. *barbadense* accessions. Red and blue boxes highlighted the candidates that positively and negatively regulated lint percentage, respectively. (**c**,**d**) Mfuzz time series of *GbPP2C* genes during different fiber development stages of high- and low-fiber-strength G. *barbadense* accessions. (**e**,**f**) Expression levels (z-scored FPKM values) of ten FL positive regulators and five FL negative regulators. (**g**) Expression levels (raw FPKM values) of a fiber strength (FS) positive regulator (*GbAIP1*).
